# A ubiquitous subcuticular bacterial symbiont of a coral predator, the crown-of-thorns starfish, in the Indo-Pacific

**DOI:** 10.1186/s40168-020-00880-3

**Published:** 2020-08-24

**Authors:** Naohisa Wada, Hideaki Yuasa, Rei Kajitani, Yasuhiro Gotoh, Yoshitoshi Ogura, Dai Yoshimura, Atsushi Toyoda, Sen-Lin Tang, Yukihiro Higashimura, Hugh Sweatman, Zac Forsman, Omri Bronstein, Gal Eyal, Nalinee Thongtham, Takehiko Itoh, Tetsuya Hayashi, Nina Yasuda

**Affiliations:** 1 Faculty of Agriculture, University of Miyazaki, 1-1 Gakuenkibanadai-Nishi, Miyazaki, Miyazaki 889-2192 Japan; 2 Biodiversity Research Center, Academia Sinica, No.128, Sec 2, Academia Rd, Nangang, Taipei, 11529 Taiwan; 3 School of Life Science and Technology, Department of Life Science and Technology, Tokyo Institute of Technology, 2-12-1 Ookayama, Meguro-ku, Tokyo, 152-8550 Japan; 4 Department of Bacteriology, Faculty of Medical Sciences, Kyushu University, 3-1-1 Maidashi Higashi-ku, Fukuoka, 812-8582 Japan; 5 Center for Information Biology, National Institute of Genetics, Yata 1111, Mishima, Shizuoka, 411-8540 Japan; 6 Australian Institute of Marine Science, PMB No.3, Townsville, QLD 4810 Australia; 7 Hawai’i Institute of Marine Biology, School of Ocean & Earth Sciences & Technology, University of Hawai’i at Mānoa, Coconut Island, Kāneʻohe, HI USA; 8 George S. Wise Faculty of Life Sciences, School of Zoology, Tel Aviv University, 6997801 Tel Aviv, Israel; 9 The Steinhardt Museum of Natural History, Israel National Center for Biodiversity Studies, Tel-Aviv University, Tel-Aviv, 6997801 Israel; 10 ARC Centre of Excellence for Coral Reef Studies, School of Biological Sciences, The University of Queensland, St. Lucia, QLD 4072 Australia; 11 The Mina & Everard Goodman Faculty of Life Sciences, Bar-Ilan University, 5290002 Ramat Gan, Israel; 12 Phuket Marine Biological Center, Botx 60, Phuket, 83000 Thailand

**Keywords:** Crown-of-thorns starfish, Subcuticular bacteria, Marine spirochetes

## Abstract

**Background:**

Population outbreaks of the crown-of-thorns starfish (*Acanthaster planci* sensu lato; COTS), a primary predator of reef-building corals in the Indo-Pacific Ocean, are a major threat to coral reefs. While biological and ecological knowledge of COTS has been accumulating since the 1960s, little is known about its associated bacteria. The aim of this study was to provide fundamental information on the dominant COTS-associated bacteria through a multifaceted molecular approach.

**Methods:**

A total of 205 COTS individuals from 17 locations throughout the Indo-Pacific Ocean were examined for the presence of COTS-associated bacteria. We conducted 16S rRNA metabarcoding of COTS to determine the bacterial profiles of different parts of the body and generated a full-length 16S rRNA gene sequence from a single dominant bacterium, which we designated COTS27. We performed phylogenetic analysis to determine the taxonomy, screening of COTS27 across the Indo-Pacific, FISH to visualize it within the COTS tissues, and reconstruction of the bacterial genome from the hologenome sequence data.

**Results:**

We discovered that a single bacterium exists at high densities in the subcuticular space in COTS forming a biofilm-like structure between the cuticle and the epidermis. COTS27 belongs to a clade that presumably represents a distinct order (so-called marine spirochetes) in the phylum *Spirochaetes* and is universally present in COTS throughout the Indo-Pacific Ocean. The reconstructed genome of COTS27 includes some genetic traits that are probably linked to adaptation to marine environments and evolution as an extracellular endosymbiont in subcuticular spaces.

**Conclusions:**

COTS27 can be found in three allopatric COTS species, ranging from the northern Red Sea to the Pacific, implying that the symbiotic relationship arose before the speciation events (approximately 2 million years ago). The universal association of COTS27 with COTS and nearly mono-specific association at least with the Indo-Pacific COTS provides a useful model system for studying symbiont-host interactions in marine invertebrates and may have applications for coral reef conservation.

Video Abstract

## Introduction

Coral reefs support almost one third of the world’s marine coastal species [1, 2]. However, population outbreaks of a coral predator, the crown-of-thorns starfish (*Acanthaster planci* sensu lato; COTS), are a great threat to Indo-Pacific coral reef ecosystem integrity and biodiversity [3–5]. A 27-year study of the Great Barrier Reef concluded that COTS outbreaks and tropical cyclones were the main causes of the loss of reef-building corals (1985–2012) [6]. While some aspects of the biology of COTS, such as its reproduction, larval ecology, phylogeography, and behaviour, have been studied intensively [5], little is known about its associated microbiota.

The bacterial symbionts of marine invertebrates have been shown to be important to their host organisms [7]. In echinoderms, bacterial communities may play a role in larval settlement [8], amino acid uptake on the integument [9], and digestive strategies in the gut [10, 11], and these communities may even drive morphological variation in their host [12]. Bacterial symbionts are prevalent on the body surfaces of echinoderms [13], showing high host specificity [14, 15]. Notably, extracellular endosymbionts known as subcuticular bacteria (SCB [16]) have been shown to reside under the cuticular layer of echinoderm fauna from all five extant classes, and it has been postulated that these bacteria provide dissolved free amino acids to their echinoderm hosts [9, 17]. To date, molecular genetic approaches targeting the 16S rRNA gene have revealed that several proteobacteria (*Alphaproteobacteria* and *Gammaproteobacteria*) are SCB that are distributed in the subcuticular space in two brittle star species [13, 18], one holothurian species [19], and one asteroid species [19].

Despite their potential biological importance, the studies of the bacteria associated with COTS have been mostly culture-based, and only two culture-independent studies have been published to date. Carrier et al. [20] reported shifts in the COTS larval microbiomes associated with diet [20]. Høj et al .[21] found that adult COTS exhibit tissue-specific bacterial communities, largely comprising four major bacterial groups: *Mollicutes* in male gonads*, Spirochaetales* in the body wall*, Hyphomonadaxeae* in the tube feet*,* and *Oceanospirillales* in all tissues [21]. Although these studies significantly increased our understanding of the COTS microbiome, there is still a great lack of knowledge regarding COTS-associated bacteria, particularly SCB, despite being common in many echinoderm taxa, where they may play an important role for their host organisms.

In the current study, we aimed to obtain primary information on the indigenous bacteria of the body surface of COTS. We carried out a comprehensive analysis of bacterial communities to find a bacterial symbiont strongly associated with COTS and examined its ubiquity using a total of 205 individuals collected from the northern Red Sea to the Pacific over a 13-year period. We highlighted the existence of dominant SCB in COTS, its novel phylogenetic status, universal distribution in the Indo-Pacific COTS, and its genomic characteristics, all of which provide insights into interactions between the COTS host and the SCB.

## Results

### Identification of a single OTU (COTS27) that dominates the body surface microbiota of COTS using 16S rRNA metabarcoding analysis

**Fig. 1:**
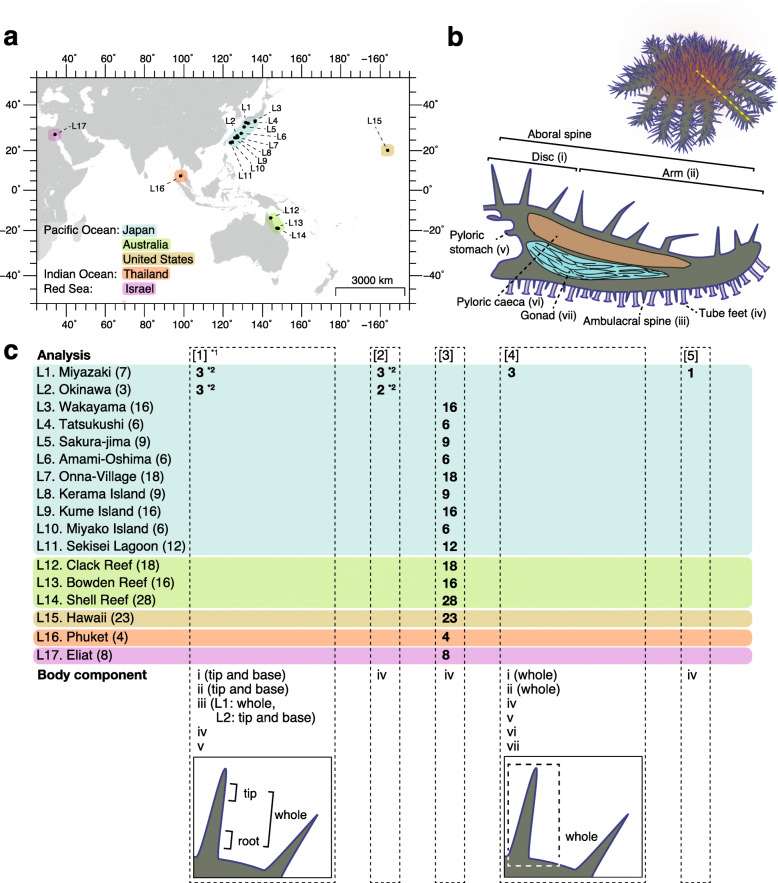
Geographic and anatomical distributions of COTS individuals and the COTS body parts analysed in this study. The seventeen locations where the COTS individuals were collected (**a**) and the dissected body parts of COTS for the analyses (**b**) are shown. The dashed yellow line (**b**) indicates the dissection line for the cross-sectional view. **c** Details of the samples used in each analysis are shown: [1] 16S rRNA metabarcoding, [2] phylogenetic analysis using the full-length 16S rRNA gene sequences, [3] PCR screening and sequencing of the 16S rRNA gene sequences of COTS27, [4] FISH analysis, and [5] hologenome sequencing analysis. *1: This analysis was performed in triplicate for each sample. *2: The same individuals were used in analyses [1, 2]

We used 16S rRNA metabarcoding to analyse the bacterial composition of the microbiota in the body parts (7–8 body parts; disc spines [top and base], arm spines [top and base], ambulacral spines [top and base for Okinawa, or the whole spine for Miyazaki], tube feet, and pyloric stomachs; Fig. 1b) of six COTS individuals that were collected in Miyazaki and Okinawa, Japan (three individuals from each location). Seawater samples from the same locations were similarly analysed for their bacterial compositions (three samples from each location). After quality filtering, 1,427,570 sequences of bacterial origins were obtained from the COTS samples (*n* = 130 for all body parts in replicates or duplicates; Suppl. table S1) and 108,334 bacterial sequences from seawater samples (*n* = 6) with averages of 10,981 and 18,056 sequences per sample, respectively (Suppl. table S2). From the abovementioned sequences, 671 bacterial OTUs were identified, 503 and 401 of which were found in the COTS and seawater samples, respectively. There were 233 OTUs that were common to both. The OTUs that were identified in the COTS and seawater samples represented bacterial taxa with 144 and 96 families, 29 and 22 OTUs were unclassified, 7 and 12 OTUs were unknown (classified as bacteria by Silva SINA [22] ), respectively (see more detail in Suppl. table S3). The rarefaction curves based on the OTUs indicated that all samples reached saturation points (Suppl. fig. S1).

**Fig. 2:**
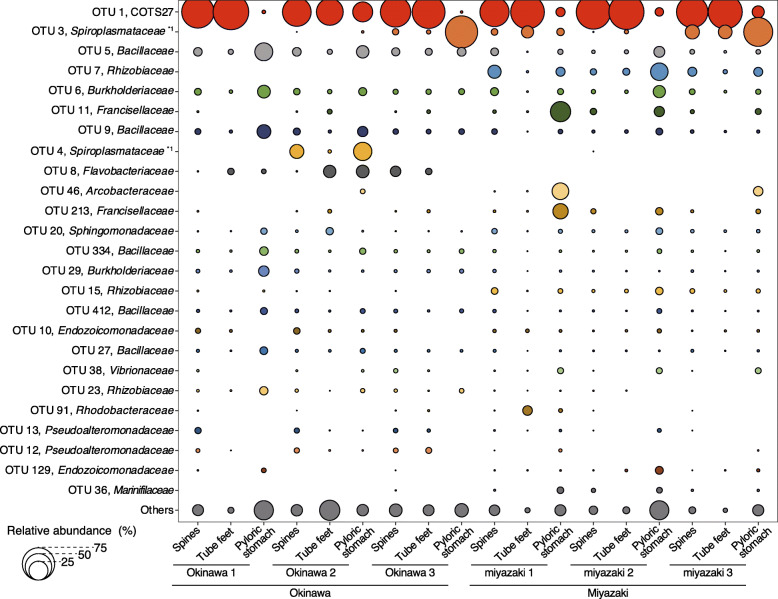
The relative abundances of the 25 most abundant OTUs, including COTS27 (OTU 1; red), in the total samples analysed in this study. The bubble chart of the relative abundances was calculated from the merged replicates of each body part (spines, tube feet, and pyloric stomachs) in each COTS individual. The phylogenies of each OTU were determined based on the results (best hit) of BLAST searches against the NCBI nr/nt database. *1: The phylogenies of OTU3 and OTU4 were determined in the All-species Living Tree Project and RDP databases, respectively

In the six COTS individuals that were examined, the relative abundance showed that a single unclassified OTU (OTU 1) occupied 61.8% of the total sequences on average, predominantly in most body parts of both the Okinawan and Miyazaki COTS populations (60.3% and 63.8% of the total sequences on average were assigned to OTU 1 in the Okinawa and Miyazaki COTS collections, respectively; Fig. 2), despite the fact that these populations were separated by more than 720 km separating these populations. The high abundance of OTU 1 in all individuals was attributed to the surface body parts (68.8% and 79.1% of the sequences from all spine and tube foot samples, respectively), with 8.0% of these sequences originating from the pyloric stomach samples (Fig. 2 and Suppl. table S4). OTU 1 was abundant at both the aboral (discs and arm spines) and oral (ambulacral spines and tube feet) sides (Suppl. fig. S2 and Suppl. table S4) of the COTS. The tips and bases of the spines showed roughly the same levels of OTU 1 abundance (Suppl. fig. S2 and Suppl. table S4). Five of the 88 spine samples that were examined (containing both tip and base) exhibited no or only a low abundance of OTU 1 (Suppl. fig. S2); however, OTU 1 was abundant in the other two DNA preparations of triplicates from the same sample in all cases, suggesting that the exceptional data from the five preparations were due to some technical problems caused by the small quantity of samples. OTU 1 was only detected in the Okinawan seawater samples, in which it showed a low abundance (0.026%; Suppl. fig. S2). The relatively abundant bacteria other than OTU 1 are described in Appendix 1. In total, we identified 41 different OTUs, including OTU 1, in all COTS individuals from the two locations, and these OTUs may represent the core members of the bacterial community of COTS (Suppl. fig. S3). The core bacterial OTUs other than OTU 1 accounted for up to 18.4% (the abundance of each OTU was less than 3.5%) of the total reads from all COTS samples (Suppl. fig. S3d). These results indicate that a single bacterium (OTU 1) predominantly colonizes the body surface of COTS.

### Phylogeny of the dominant OTU 1 (COTS27) based on 16S rRNA gene sequences

**Fig. 3:**
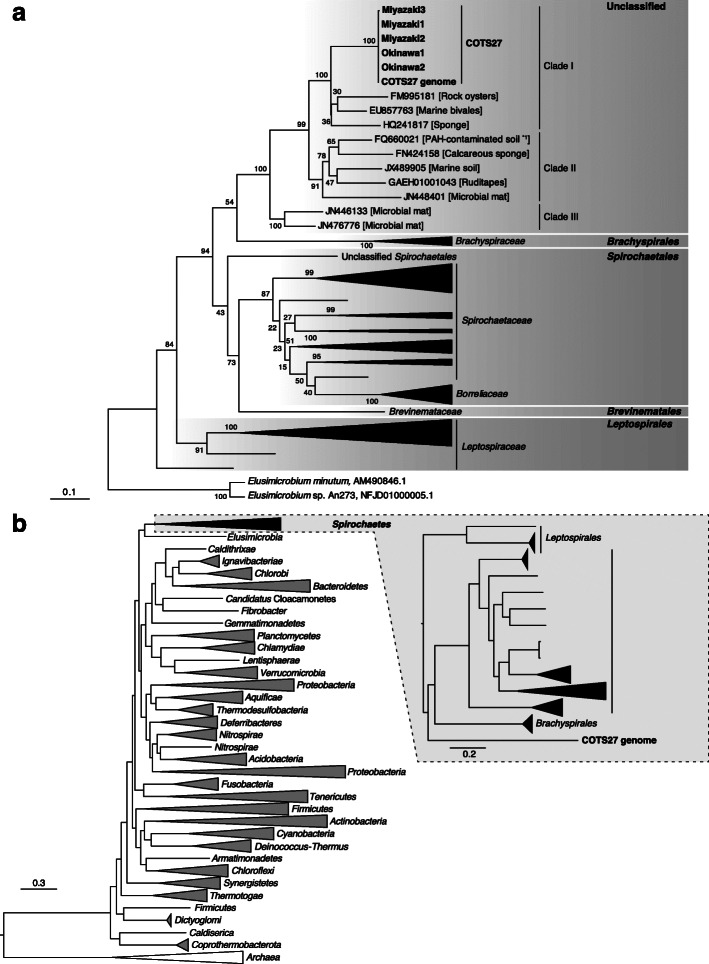
The phylogenetic position of the dominant OTU 1 (COTS27) in the phylum *Spirochaetes*. Maximum likelihood (ML) trees were constructed based on the full-length 16S rRNA gene sequences (**a**) and the sequences of 43 conserved marker genes identified by CheckM (**b**). The bootstrap values in **a** were calculated by resampling 1000 times. The scale bars indicate substitutions per site. *1: The gene with accession No. FQ660021.1 in **a** was obtained from a polycycle aromatic hydrocarbon (PAH)-contaminated soil sample in a mitigated wetland.

To elucidate the phylogenetic status of the dominant OTU 1, we determined the full-length 16S rRNA gene sequences of OTU 1 in five tube foot samples obtained from Miyazaki (*n* = 3) and Okinawa (*n* = 2). The five sequences were largely identical (99.9–100% similarity), and there was a partial sequence overlap with the 16S rRNA gene sequence of a spirochete-like bacterium (GenBank accession No. PRJNA420398) that was a dominant bacterium on the body wall of COTS from the Great Barrier Reef [21]. The maximum likelihood (ML) phylogenetic tree (Fig. 3a) based on full-length 16S rRNA gene sequences showed that the five sequences related to the OTU 1 formed a distinct subclade within one of the three clades of the unclassified spirochete cluster (named clade I; Fig. 3a). All sequences in this unclassified spirochete cluster originated from marine environments and marine invertebrates (see Appendix 2 for more details of clade I) with the exception of a single sequence obtained from a wetland soil sample (GenBank accession No. FQ660021.1). Hereafter, we refer to these spirochetes as “marine spirochetes”, as referred to by Høj et al. [21]. These marine spirochetes formed a distinct cluster within the phylum *Spirochaetes*, with the order *Brachyspirales* being their closest relative (Fig. 3a). Notably, the 16S rRNA gene sequences of the marine spirochetes, including the OTU 1 group, showed only a 76.3–78.1% identity to those of the order *Brachyspirales*, which is well below the proposed threshold for defining a novel order (82.0%) [23]. Thus, the marine spirochetes most likely represent a distinct order in the phylum *Spirochaetes*. Hereafter, we refer to the bacterium corresponding to the OTU 1 as COTS27.

### Universal association of COTS27 with COTS throughout the Indo-Pacific Ocean

The presence of COTS27 or COTS27-like bacteria in COTS individuals inhabiting various geographic regions was determined in a PCR assay designed to amplify a specific 261 bp fragment of the COTS27 16S rRNA gene. PCR products were obtained from all 195 COTS individuals that were collected at 15 locations throughout the Indo-Pacific Ocean comprising three known species of COTS (Fig. 1a, c). The sequencing of the PCR products from 53 randomly selected individuals confirmed the presence of COTS27 or very close relatives. The ML tree based on these 261 bp sequences (Suppl. fig. S4) revealed that all sequences formed a tight cluster with the six COTS27 sequences from the abovementioned phylogenetic analysis and with those obtained from the genome reconstruction described below. However, the sequences from the Israeli COTS population (Red Sea species) formed a clade separate from those of the Indo-Pacific populations from the northern Indian Ocean or Pacific Oceans. Among the northern Indo-Pacific species, only one single-nucleotide polymorphism (SNP) was detected in one sequence obtained from Japan (Wakayama C29 adult JPN; Suppl. fig. S4). These results indicate the universal association of COTS27 with the Indo-Pacific COTS species.

### Localization and biofilm-like structure formation of COTS27 in subcuticular spaces across the body surface of COTS

**Fig. 4:**
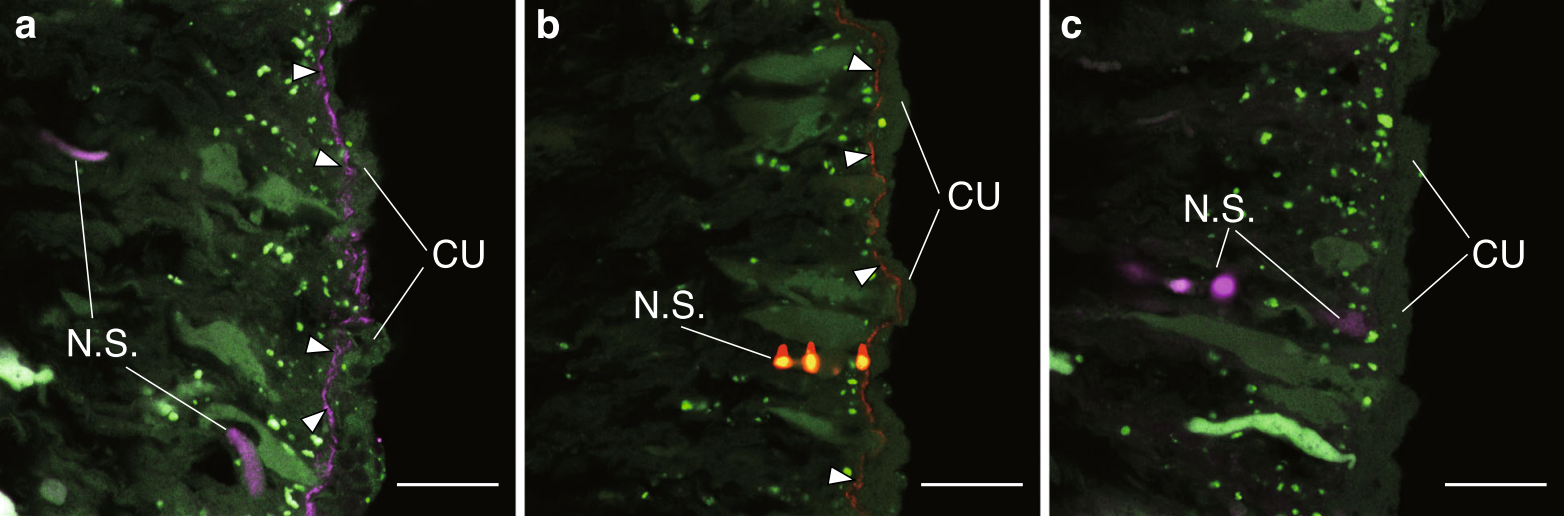
FISH analysis of three serial sections of a COTS disc spine. Each section was hybridized with the EUB338mix (**a**, purple; a general probe for bacteria), COTSsymb (**b**, red; a COTS27-specific probe), or Non338 (**c**, purple; a negative control to detect non-specific binding) probes. The probes were labelled with Cy3 in all panels and coloured with purple in **a** and **c** and with red in **b**. The green signals are tissue-derived autofluorescence. The arrowheads in **a** and **b** indicate layer-like signals from the general probe for bacteria (**a**) and the COTS27- specific probe (**b**). N.S. and C.U. indicate regions with non-specific binding and the outer cuticle complex, respectively. The scale bars represent 20 μm (**a**–**c**)

**Fig. 5:**
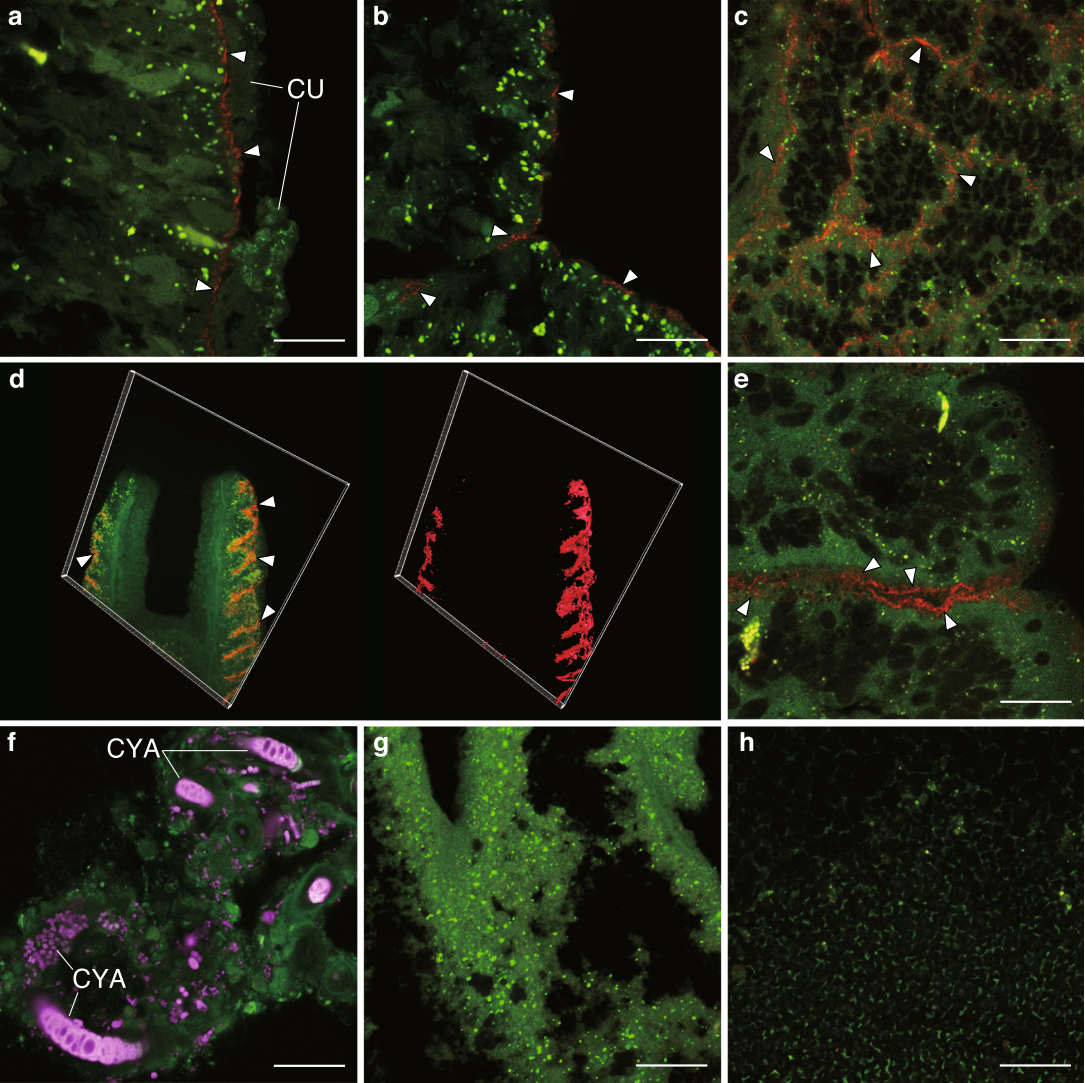
Visualization of the COTS27 cells in different body parts of COTS using FISH. COTS27 cells (red) residing in the subcuticular spaces of the body walls were detected with COTSsymb, a COTS27-specific probe, in the tips (**a**) and bases (**b**) of aboral spines on the discs and arms, respectively, dermal papula (**c**), pedicellariae on the aboral side (**d**; 3D image [left] and 3D rendering image [right]), and tube feet (**e**). Many non-COTS27 bacteria (purple) were detected in the pyloric stomachs (**f**) using the EUB338mix probe. No visible bacteria were detected in the pyloric caeca (**g**) and gonads (**h**) in this study (the images were obtained applying the COTS27-specific probe). The arrowheads indicate signals from COTS27. The green signals are tissue-derived autofluorescence. CU, outer cuticle complex; CYA, cyanobacteria-like cells. Scale bars (**a–c** and **e–h**) indicate 20 μm. The 3D image in panel **d** was taken with an original objective of × 40

We observed the localization of COTS27 in COTS tissues using fluorescence in situ hybridization (FISH), as demonstrated by the binding of the general bacterial probe and a COTS27-specific probe that we designed (the binding signals on the COTS central disc spines are shown in Fig. 4). COTS27 was consistently present in the subcuticular spaces on both the aboral side (Fig. 5a–d; spines of the discs and arms, dermal papulae, and pedicellariae, see Fig. 1b and Suppl. fig. S5 for their anatomical locations and structures) and oral side (Fig. 5e; the stems of the tube feet) and the pattern was similar for all three COTS individuals (Figs. 4 and 5). No COTS27 signal or any other bacterial signals were detected in the pyloric caeca and gonads (Fig. 5g, h). Likewise, no COTS27 was found in the pyloric stomachs, although numerous cyanobacteria-like bacteria were detected (Fig. 5f).

In the cross-sections, COTS27 displayed continuous layer-like signals (Figs. 4b and 5a–c, e), although a patchy distribution was also occasionally observed. Furthermore, three-dimensional (3D) images showed that COTS27 formed a biofilm-like structure on the epidermis of the pedicellariae (Fig. 5d). These observations indicate that COTS27 is an SCB that covers nearly all the surface area (the epidermis) of COTS by forming a biofilm-like structure. COTS27 cells appear to have filamentous or long rod-like shapes (Figs. 5c, e), but different approaches such as electron microscopy are required to accurately determine their cell morphology.

### Reconstruction of the COTS27 chromosome

**Fig. 6:**
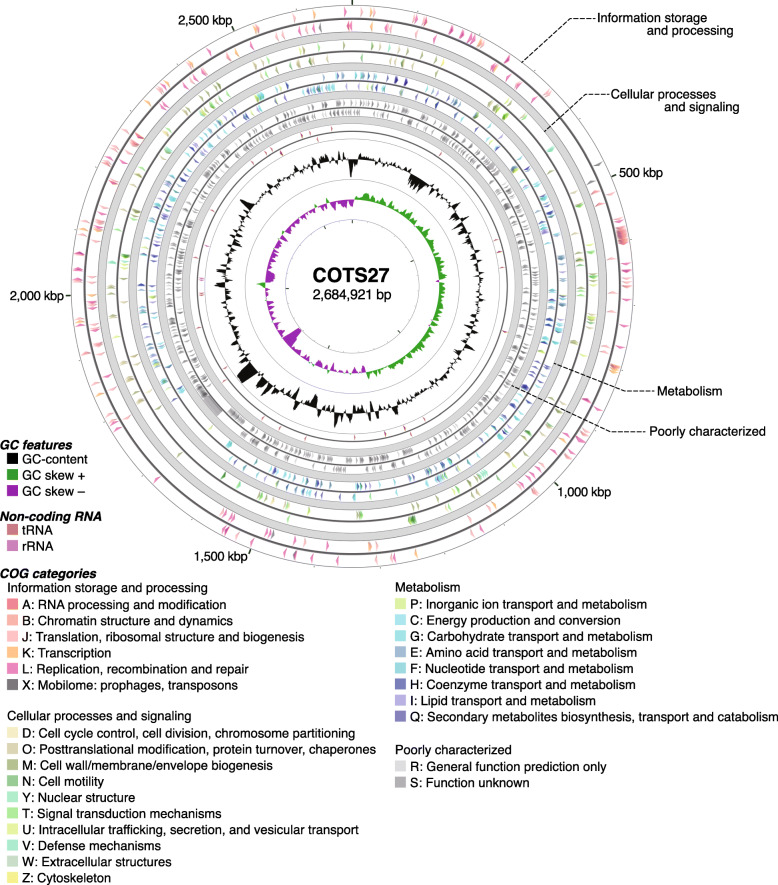
Circular view of the COTS27 chromosome. From the outside to the centre, each circle indicates forward strand CDSs, reverse strand CDSs, forward strand tRNA and rRNA genes, reverse strand tRNA and rRNA genes, GC-content, and GC skew. The CDSs were coloured according to the COG functional category of each CDS. The circular maps were created using CGView Server and the designations were then superposed manually

We have not yet succeeded in isolation of COTS27, but were able to reconstruct the chromosome sequence of COTS27 from the hologenome sequences, which contained sequences derived from the host genome and the associated microbes (Suppl. table S5), of a COTS sample collected in Miyazaki (Fig. 6 and Suppl. table S6), with 90.66% completeness and 0.26% contamination, as evaluated by CheckM [24]. The structural accuracy of the chromosome was validated based on the physical coverage of the 15 kbp-mate-pairs (Suppl. Materials and Methods Fig. 1), and the circular structure was also confirmed using PCR and Sanger sequencing. We also tested other assembly pipelines consisting of removal of reads from the host genome, metagenome assemblers, and binning tools; however, none generated a higher-quality genome of COTS27 nor an obvious chromosome of a different bacterium (Suppl. Materials and Methods table 1). Although 23 gaps remained in the final assembly, all were derived from tandem repeats in the genic regions, and the estimated gap sizes were less than 28 bp. The COTS27 chromosome was 2,684,921 bp in length, with a 39.6% average GC-content, and contained 1650 protein-coding genes, three rRNA genes, and 35 tRNA genes. No transposable elements or prophages were detected. The 1650 protein-coding genes included one giant gene (53,043 bp in length; COTS27_01023), but its function is currently unpredicted (see Suppl. materials and methods for the details). Among the three rRNA genes, the 16S rRNA gene was located separately from the 23S and 5S rRNA genes. The 35 tRNA genes covered all 20 basic amino acids. Phylogenetic analysis using the sequences of 43 conserved marker genes with 5656 reference bacterial and archaeal genomes placed COTS27 in the phylum *Spirochaetes* (Fig. 3b), supporting the results of the 16S rRNA sequence-based analysis (Fig. 3a).

### Biological features of COTS27 inferred from the gene repertoire

In the Clusters of Orthologous Groups (COG) functional category-based principal component analysis of COTS27 performed using 716 high-quality *Spirochaetes* genomes obtained from the IMG database [25], COTS27 was placed in a distinct position with regard to all *Spirochaetes* (Suppl. fig. S6). This indicates potential biological features unique to COTS27. Subsequently, we performed a Kyoto Encyclopedia of Genes and Genomes (KEGG) pathway analysis to obtain basic information on the biology of COTS27 (Suppl. table S7). Complete or near-complete biosynthesis pathways for 18 of the 20 basic amino acids were identified, excluding those for asparagine and aspartic acid. Although the guanine ribonucleotide biosynthesis pathway was not complete (one block missing), all other nucleotide biosynthesis pathways were detected. For vitamin and cofactor biosynthesis, the complete biosynthetic pathways of nicotinamide adenine dinucleotide (NAD), coenzyme A, and riboflavin and the C1-unit interconversion were identified. Pathways for fatty acid biosynthesis, beta-oxidation, and phosphatidylethanolamine biosynthesis were also detected. The conservation of these metabolic pathways suggests that COTS27 is not strongly metabolically dependent on the host COTS.

Regarding energy production, COTS27 exhibited the complete glycolysis pathway and TCA cycle. Genes for succinate dehydrogenase, cytochrome c oxidase, and F-type ATPase were also identified; however, no genes for NADH dehydrogenase were detected. Instead, COTS27 presented an operon encoding a sodium-pumping NADH:ubiquinone oxidoreductase (Na^+^-NQR) (Suppl. fig. S7).

Consistent with the general characteristics of *Spirochaetes*, which are generally Gram-negative, helical or spiral-shaped, and motile, with periplasmic flagella [26], COTS27 contained sets of genes for the biosynthesis of DAP-type peptidoglycan, lipopolysaccharide, and phosphatidylethanolamine. While a set of genes for flagellar biosynthesis was identified, no gene for chemotaxis (such as genes encoding methyl-accepting chemotaxis proteins and chemotaxis-related signal transduction components) was detected.

## Discussion

We identified a single bacterium that forms a subcuticular biofilm-like structure in Indo-Pacific COTS although we cannot completely exclude the possibility that the signals detected in FISH analyses included some false-positive signals that may require additional verification. This bacterium is universally present and numerically dominant and likely represents a previously undefined order within the phylum *Spirochaetes*. The universal association of COTS27 with the Indo-Pacific COTS species suggests a long history of the COTS-COTS27 association. COTS are thought to have allopatrically diverged into four species during the Pliocene-Early Pleistocene (1.95–3.65 Myr ago) in the Indo-Pacific Ocean [27]. Therefore, the association of COTS27 with at least three of the four extant COTS species (data for the fourth species are currently not available) implies that the mutual relationship between COTS and COTS27 emerged prior to the Pliocene or Early Pleistocene eras. This hypothesis is supported by the finding that COTS27 from the northern Red Sea (forming a different cluster from the Indo-Pacific regions; Suppl. fig. S4) was notably different from COTS27 from other regions. Additional comparative genomic analyses of the Indo-Pacific COTS and COTS27 from different geographic regions would provide more detailed insights into the possible co-evolutionary history. In addition, further studies linking environmental conditions with COTS27 abundance and microbial composition will help to understand the ecological roles of COTS27.

Regarding the evolutionary and functional aspects of COTS27 and its association with COTS, there were two key findings from the genome sequence analysis: (1) the presence of Na^+^-NQR and (2) the selective loss of chemotaxis genes. Some bacteria living in Na^+^-rich environments (e.g., marine or intercellular environments) exhibit a Na^+^-NQR that oxidizes NADH to NAD^+^ and pumps Na^+^ out of cells; thus, functioning in the respiratory chain and in the maintenance of intercellular homeostasis in Na^+^-rich environments [28]. With regard to Na^+^-NQR, only one genome out of the 716 high-quality *Spirochaetes* genomes (above), which was also reconstituted from the metagenome sequences of a seawater sample (*Spirochaetaceae* bacterium NP120, IMG Genome ID 2509276057), contained the Na^+^-NQR operon. The acquisition of Na^+^-NQR may therefore represent one of the mechanisms responsible for the adaptation of COT27 to marine environments. With regard to the selective loss of chemotaxis genes, it is very unusual in *Spirochaetes*; most of the high-quality *Spirochaetes* genomes mentioned above (> 98%) contained gene sets for both flagellar biosynthesis and chemotaxis. The remaining genomes, such as those from the genus *Sphaerochaeta*, lack genes for both flagellar biosynthesis and chemotaxis, suggesting that chemotaxis genes have been selectively lost from the COTS27 genome. It has been proposed that the active migration and colonization by symbionts through motility and chemotaxis are often required for the acquisition of microbial partners by host organisms from their environments [29]. However, the selectively lost chemotaxis genes appear to represent a specific adaptation strategy of COT27 as an SCB. COTS27 may require flagella to spread widely and to stably colonize subcuticular spaces, but chemotaxis is not likely to be required after adapting and specializing to the subcuticular spaces of COTS. These findings are informing because these features are likely linked to the adaptation to marine environments and evolution as an extracellular endosymbiont in the subcuticular space. Additional genome sequences of marine spirochetes are required to verify this hypothesis and elucidate the evolutionary and functional aspects of the COTS27-COTS association.

Our study implied that COTS27 as SCB forms a nearly mono-specific symbiotic relationship with COTS, at least with the Indo-Pacific COTS. SCB, however, are commonly found in echinoderm fauna [13, 19] and have been classified into three major morphotypes [13, 15, 16]. Among these morphotypes, COTS27 most likely belongs to the SCB Type 2, which exhibits a long spiral shape and is commonly found among all five echinoderm classes [15, 30]. Jackson et al. [31] suggested the presence of a highly dominant *Spirochaetae* in the hard tissues (including the body wall) of some starfish species in the USA and Australia [31]. Such a wide distribution of spirochetes or spirochete-like bacteria in echinoderms suggests that many echinoderms may have established symbiotic relationships with marine spirochetes that are similar to that between COTS27 and COTS. Further explorations of SCB in a wider range of echinoderms would provide more detailed insights into the association between echinoderm hosts and marine spirochetes.

The outer body surfaces of marine organisms often represent a highly active interface between an organism (host) and the surrounding marine environment regarding aspects such as light exposure, gas exchange, nutrient uptake, and interactions with other fouling organisms, consumers, and pathogens [32]. The presence of SCB among different echinoderms has been reviewed in different bacterial taxa [13, 18, 19]. Although it is also plausible that SCB play hypothetical role in their interactions with the host such as nutrition transfer [9, 33], or antibiotics production [14, 34], or even that their presence may be vestigial, remaining as leftovers from a previously mutualistic partnership [15], the physiological and potential ecological roles of SCB are largely unclear and remain unexplored. The universal and nearly mono-specific association of COTS27 with COTS would provide an ideal model system for further exploring the roles of SCB as well as symbiont-host interactions in marine invertebrates. Moreover, COTS27 could be used as an environmental marker to monitor and/or predict population outbreaks of COTS.

## Conclusions

Despite the fact that the 205 COTS individuals utilized in our current analyses were collected over a 13-year period (2004–2017) and from 17 different locations across the Indo-Pacific, the COTS27 association remained exceptionally ubiquitous both spatially and temporally. Additionally, it is likely that COTS hosted COTS27 as an extracellular endosymbiont for more than 2 million years before allopatric speciation occurred during the Pliocene-Early Pleistocene suggesting a strong association. COTS27 is likely an extracellular endosymbiotic bacteria strongly associated with COTS as an SCB. COTS27 also would acquire the Na^+^-NQR system for adapting to the marine environment since speciation within the phylum *Spirochaetes.* The lack of chemotaxis genes in COTS27 may have resulted from specialization for the unique habitat of the subcuticular space of COTS. Although the functional role of COTS27 as an SCB is still unclear, this close relationship and genomic characterization of COTS27 described here will significantly contribute to testing the hypotheses of symbiotic function in SCB, and may also provide as a model system for studying endosymbionts in marine invertebrates more broadly.

## Materials and methods

### Sample collection and preparation for DNA analyses and histology

We collected 205 individual COTS from 17 locations throughout the Indo-Pacific collected over a 13-year period (2004–2017; Fig. 1a, and Suppl. table S8). For 16S rRNA metabarcoding, six individuals were collected in Okinawa and Miyazaki (three from each location) in Japan (Fig. 1a). The specimens were dissected to facilitate sampling from different body parts (7–8 body parts; Fig. 1b, c) which was done in triplicate or duplicate (Suppl. table S1). Seawater samples were also collected in triplicate from each of the last two locations. Consequently, 130 DNA samples were prepared from the six COTS individuals (Suppl. table S1) and six seawater DNA samples for the metabarcoding analysis. The tube foot DNA samples from five of the six individuals were used to determine the full-length 16S rRNA gene sequence of the dominant OTU 1. DNA samples prepared from the tube feet of 195 individuals collected from 15 geographic locations were used to examine the presence of COTS27 (the dominant OTU 1) in three species of COTS [35] (Fig. 1a, c, and Suppl. table S8). DNA from the tube feet of one individual collected in Miyazaki (Japan) was used for the COTS hologenome sequencing (Suppl. table S8) [36].. Note that the experiment was designed to capture both the host and associated microbiome genomes as a hologenome. Samples of six different body parts (Fig. 1b, c) were prepared from the remaining three individuals collected in Miyazaki for the FISH analyses.

We provide a more detailed description of the above collection and preparation methods in the Suppl. materials and methods.

### 16S rRNA metabarcoding

16S rRNA amplicon libraries (V4 region) were prepared as previously described [37, 38] using the primers listed in Suppl. table S9 and Suppl. fig. S8 and subjected to paired-end (PE) sequencing (2 × 300 bp) using the Illumina MiSeq platform. In total, 130 DNA samples including the samples collected from 7 to 8 body parts of six COTS individuals (Suppl. table S1), and six seawater DNA samples were analysed. The obtained PE sequences were processed using software USEARCH v8.1.1861 [39] and MOTHUR v.1.36.1 [40] software for the merging, the filtering, the OTU clustering, and the taxonomic assignment of the sequence (see more detail in Suppl. materials and methods). Finally, a total of 1,535,904 sequences were assigned to bacteria. The others were assigned to eukaryotes (55,377 reads containing 74.7% of COTS genes), Archaea (12,686 reads), chloroplasts (19,715 reads), or unknown origins (476,795 reads containing 99.4% of COTS genes) and were excluded from our study (Suppl. table S2).

### Phylogenetic analysis of OTU 1 using the full-length 16S rRNA gene sequence

Full-length 16S rRNA gene sequences of OTU 1 were obtained from each tube foot of five COTS individuals using a specific primer set for OTU 1 that was designed in this study (Suppl. materials and methods, Suppl. table S9, and Suppl. fig. S8). The sequences were used to reconstruct the phylogenetic tree using the maximum likelihood (ML) method (Suppl. materials and methods). As OTU 1 was revealed to represent a unique clade of bacteria present in COTS, we hereafter refer to this bacterium as COTS27.

### PCR screening and sequencing of COTS27

In total, 195 COTS individuals were screened for the presence of COTS27 on their tube feet by PCR using primers that were designed to specifically amplify a 261 bp fragment of the 16S rRNA gene (Suppl. table S9 and Suppl. fig. S8). The PCR products obtained from 53 randomly selected samples from all COTS27-positive samples (*n* = 195) were sequenced and used for phylogenetic reconstruction (Suppl. materials and methods).

### Fluorescence in situ hybridization (FISH)

The FISH experiments were performed on three serial sections (thickness of 5 μm) from the six body parts of the three individuals (Fig. 1b, c) as previously described [41]. FISH was performed separately with three different probes: COTS27-specific oligonucleotide probe (COTSsymb; for more detail of the probe design, see in Suppl. materials and methods, Suppl. table S9, and Suppl. fig. S8), a Eubacterial probe (EUB338mix [42]), and a nonsense probe (Non338 [43]). Bacterial localization was observed using a confocal laser scanning microscope (LSM 550; Zeiss, Germany) (see more detail in Suppl. materials and methods). In addition, we reconstructed three-dimensional (3D) structures from thick sections (thickness, 50 μm) of the disc spines using a confocal laser scanning microscope (LSM770; Zeiss, Germany),

### Reconstitution of the COTS27 chromosome from the hologenome sequences of a COTS sample

Two PE libraries and six mate-pair libraries from the tube foot of one individual were prepared and sequenced using Illumina HiSeq 2500 sequencers. De novo assembly was performed using Platanus v. 1.2.3 [44], resulting in 95,349 scaffolds whose total size was 440,015,850 bp. The full-length 16S sequence of COTS27 was searched to the assembly by BLASTN, and one scaffold (length, 2,331,938 bp; PE coverage depth, × 210) was hit (identity, 100%; alignment length, 1384 bp). The average PE coverage depth for all the scaffolds was calculated as × 130, and we assumed that the sequences of the COTS27 genome had higher coverage depth compared to the host COTS and other minor microbes. Since the PE libraries were prepared using the TruSeq PCR-free kit, this situation may reflect that the number of cells of COTS27 was greater than that of the host. Additionally, we confirmed that the majority of the scaffolds (83%) indicated a similarity to the COTS reference genome (tool, BLASTN; e-value cut-off, 10^−20^). To identify all the COTS27-derived sequences in the hologenome assembly, scaffolds with high coverage depths (≥ × 200) were selected. To extend the selected ones, an additional scaffolding process was performed using Platnaus-allee v. 2.0.0 [45]. The longest scaffold (2,723,166 bp) in the result, which contained the 16S sequence of COTS27, was identified as the COTS27 chromosome. Finally, gaps in the chromosome were closed by Sanger sequencing and an alternative assembly generated by Platanus-allee v. 2.0.0 [45]. Here, the circular structure of the chromosome was also confirmed.

A circular view of the COTS27 chromosome was generated using the CGView Server [46] with manual processing. The completeness of the final assembly was evaluated using CheckM v. 1.0.11 [24], and the structural accuracy of the assembly was validated based on the physical coverage of the 15 kbp-mate-pairs (see Suppl. materials and methods for the details). We also tested other assembly pipelines consisting of removal of reads from the host genome, metagenome assemblers, and binning tools (see Suppl. materials and methods for details).

### Gene prediction and functional annotation

Protein-coding sequences (CDSs) were predicted by using PROKKA v. 1.12 [47], followed by manual curation. For functional annotation, Clusters of Orthologous Groups (COG) were assigned by querying the CDSs against the Conserved Domain Database (CDD) with COG position-specific scoring matrices (PSSMs) using RPS-BLAST. Additionally, K numbers of Kyoto Encyclopedia of Genes and Genomes (KEGG) were assigned to each CDS; BlastKOALA [48], and KofamKOALA [49] were used to perform searches in the KEGG GENES and KOfam databases, respectively.

### Principal component analysis and phylogenetic analysis based on the genome sequences

Principal component analysis was performed based on the compositions of the COG functional categories. The genome sequences of the *Spirochaetes* bacteria were retrieved from the DOE-JGI IMG database, and 716 high-quality genomes (completeness > 90% and contamination < 5% as evaluated by CheckM v. 1.0.11 [24]) were retained (see Suppl. materials and methods for the details). Whole-genome sequence-based phylogenetic analysis was performed using CheckM to obtain the ML tree of COTS27 and 5656 bacterial and archaeal genomes based on the sequences of 43 conserved marker genes. The tree was visualized using FigTree v. 1.4.3 (http://tree.bio.ed.ac.uk/software/figtree/).

## Supplementary information

**Additional file 1: Suppl. Table S1.** Sample number of body parts from COTS in the 16S rRNA metabarcoding analysis (total 130 samples)

**Additional file 2: Suppl. Table S2.** Number of OTUs and sequence reads obtained from the 16S rRNA metabarcoding

**Additional file 3: Suppl. Table S3.** Bacterial taxa number identified from phylum to family in the COTSs and seawater samples (total 761 OTUs). **Suppl. Table S4.** Abundance of COTS27 (OTU1) in the different body components of COTS among Okinawa and Miyazaki samples. **Suppl. Table S5.** COTS genome sequencing read data. All reads were preprocessed to exclude adaptor sequences and low-quality bases by Platanus_trim (version 1.0.7; http://platanus.bio.titech.ac.jp/). For all mate-pair libraries, short-insert pairs (estimated insert size ≤ 0.5 × nominal size) and PCR products, duplicates were removed based on mapping information to the assembled results (scaffolds), which were constructed only from the paired-end libraries, using an in-house program. **Suppl. Table S6.** COTS27 genome information. **Suppl. Table S7.** COTS27 genome general biosynthesis profile based on KEGG metabolic pathways. **Suppl. Table S8.** Samples used for 16S rRNA metabarcoding, reconstruction of phylogenetic tree, and PCR screening and sequencing. **Suppl. Table S9.** Primers used for 16S rRNA gene sequence analysis and probes for FISH used in this study

**Additional file 4: Supplementary figures. Suppl. Fig. S1.** Rarefaction curves of bacterial OTUs from COTS and seawater. **Suppl. Fig. S2.** Stacked bar-plots of the relative abundance of bacterial OTUs. **Suppl. Fig. S3.** Bacterial OTUs shared by COTS individuals. **Suppl. Fig. S4.** Phylogenetic analysis of COTS27 obtained from 59 COTS individuals. **Suppl. Fig. S5.** Schematic drawing showing the dermal papula (skin gill) and pedicellariae (small external appendages) between disc spines (b) on the body surface of COTS (a). **Suppl. Fig. S6.** Principal component analysis (PCA) of COTS27 and 716 high-quality Spirochaetes genomes based on the gene abundance according to the COG functional categories. **Suppl. Fig. S7.** Gene organization and position of the Na+-NQR genes on the COTS27 chromosome. **Suppl. Fig. S8.** Positions of the primers and probes used in the current study for the 16S rRNA gene analysis

**Additional file 5: Appendixes Appendix 1.** Other relatively abundant bacteria in COTS. **Appendix** **2.** The members of clade I in marine spirochetes

**Additional file 6: Supplementary materials and methods.**

## Data Availability

All sequences produced for this study have been deposited in the DDBJ under BioProject accession number PRJDB4009 for the 16S metabarcoding and COTS27 chromosome data and accession numbers LC490103–LC490107 and LC495323–LC495375 for the 16S rRNA gene sequence-based phylogeny.

## Abbreviations

CDD: Conserved Domain Database
CDSs: Protein-coding sequences
COG: Clusters of Orthologous Groups
COTS: Crown-of-thorns starfish
FISH: Fluorescence in situ hybridization
KEGG: Kyoto Encyclopedia of Genes and Genomes
ML: Maximum likelihood
NAD: Nicotinamide adenine dinucleotide
Na+-NQR: Sodium-pumping NADH:ubiquinone oxidoreductase
OTU: Operational taxonomic unit
SCB: Subcuticular bacteria

## References

[1] Spalding MD, Ravilious C, Green EP. World atlas of coral reefs. Prepared at the UNEP World Conservation Monitoring Centre. Univ Calif Berkeley EEUULinks. 2001;.

[2] Wilkinson C. Status of coral reefs of the world: 2004. Australian Institute of Marine Science; 2004.

[3] Moran P, Bradbury R (1989). The crown-of-thorns starfish controversy. Search..

[4] Birkeland C, Lucas J. *Acanthaster planci*: major management problem of coral reefs. CRC Press; 1990.

[5] Pratchett MS, Caballes CF, Sweatman JAR-P& HPA (2014). Limits to understanding and managing outbreaks of crown-of-thorns starfish (*Acanthaster* spp.). Oceanogr Mar Biol.

[6] De’ath G, Fabricius KE, Sweatman H, Puotinen M (2012). The 27–year decline of coral cover on the Great Barrier Reef and its causes. Proc Natl Acad Sci.

[7] McFall-Ngai M, Hadfield MG, Bosch TCG, Carey HV, Domazet-Lošo T, Douglas AE (2013). Animals in a bacterial world, a new imperative for the life sciences. Proc Natl Acad Sci.

[8] Bryan PJ, Rittschof D, McClintock JB (1996). Bioactivity of echinoderm ethanolic body-wall extracts: an assessment of marine bacterial attachment and macroinvertebrate larval settlement. J Exp Mar Biol Ecol.

[9] Lesser MP, Walker CW (1992). Comparative study of the uptake of dissolved amino acid in sympatric brittle stars with and without endosymbiotic bacteria. Comp Biochem Physiol Part B Comp Biochem.

[10] Thorsen MS (1999). Abundance and biomass of the gut-living microorganisms (bacteria, protozoa and fungi) in the irregular sea urchin *Echinocardium cordatum* (Spatangoida: Echinodermata). Mar Biol.

[11] Thorsen MS, Wieland A, Ploug H, Kragelund C, Nielsen PH (2003). Distribution, identity and activity of symbiotic bacteria in anoxic aggregates from the hindgut of the sea urchin *echinocardium cordatum*. Ophelia..

[12] Balakirev ES, Pavlyuchkov VA, Ayala FJ (2008). DNA variation and symbiotic associations in phenotypically diverse sea urchin *Strongylocentrotus intermedius*. Proc Natl Acad Sci.

[13] Burnett WJ, McKenzie JD (1997). Subcuticular bacteria from the brittle star *Ophiactis balli* (Echinodermata: Ophiuroidea) represent a new lineage of extracellular marine symbionts in the alpha subdivision of the class *Proteobacteria*. Appl Environ Microbiol.

[14] McKenzie JD, Kelly MS (1994). Comparative study of sub-cuticular bacteria in brittlestars (Echinodermata: Ophiuroidea). Mar Biol.

[15] Kelly MS, McKenzie JD (1995). Survey of the occurrence and morphology of sub-cuticular bacteria in shelf echinoderms from the north-east Atlantic Ocean. Mar Biol.

[16] Holland ND, Nealson KH (1978). The fine structure of the echinoderm cuticle and the subcuticular bacteria of echinoderms. Acta Zool.

[17] McKenzie JD, Black KD, Kelly MS, Newton LC, Handley LL, Scrimgeour CM (2000). Comparisons of fatty acid and stable isotope ratios in symbiotic and non-symbiotic brittlestars from Oban Bay, Scotland. J Mar Biol Assoc U K.

[18] Morrow KM, Tedford AR, Pankey MS, Lesser MP (2018). A member of the Roseobacter clade, *Octadecabacter* sp., is the dominant symbiont in the brittle star *Amphipholis squamata*. FEMS Microbiol Ecol.

[19] Lawrence SA, O’Toole R, Taylor MW, Davy SK (2010). Subcuticular bacteria associated with two common New Zealand echinoderms: characterization using 16S rRNA sequence analysis and fluorescence in situ hybridization. Biol Bull.

[20] Carrier TJ, Wolfe K, Lopez K, Gall M, Janies DA, Byrne M (2018). Diet-induced shifts in the crown-of-thorns (*Acanthaster* sp.) larval microbiome. Mar Biol.

[21] Høj L, Levy N, Baillie BK, Clode PL, Strohmaier RC, Siboni N (2018). Crown-of-Thorns sea star *Acanthaster* cf. *solaris* has tissue-characteristic microbiomes with potential roles in health and reproduction. McBain AJ, editor. Appl Environ Microbiol.

[22] Pruesse E, Peplies J, Glöckner FO (2012). SINA: accurate high-throughput multiple sequence alignment of ribosomal RNA genes. Bioinformatics..

[23] Yarza P, Yilmaz P, Pruesse E, Glöckner FO, Ludwig W, Schleifer K-H (2014). Uniting the classification of cultured and uncultured bacteria and archaea using 16S rRNA gene sequences. Nat Rev Microbiol.

[24] Parks DH, Imelfort M, Skennerton CT, Hugenholtz P, Tyson GW (2015). CheckM: assessing the quality of microbial genomes recovered from isolates, single cells, and metagenomes. Genome Res.

[25] Chen I-MA, Chu K, Palaniappan K, Pillay M, Ratner A, Huang J (2019). IMG/M v.5.0: an integrated data management and comparative analysis system for microbial genomes and microbiomes. Nucleic Acids Res.

[26] Paster BJ. Phylum XV. Spirochaetes Garrity and Holt 2001. In: Krieg NR, Staley JT, Brown DR, Hedlund BP, Paster BJ, Ward NL, et al., editors. Bergey’s Manual® syst bacteriol vol four bacteroidetes spirochaetes tenericutes mollicutes acidobacteria fibrobacteres fusobacteria dictyoglomi gemmatimonadetes lentisphaerae verrucomicrobia chlamydiae planctomycetes. New York, NY: Springer New York; 2010. p. 471–566.

[27] Vogler C, Benzie J, Lessios H, Barber P, Wörheide G (2008). A threat to coral reefs multiplied? Four species of crown-of-thorns starfish. Biol Lett.

[28] Reyes-Prieto A, Barquera B, Juárez O (2014). Origin and evolution of the sodium-pumping NADH: ubiquinone oxidoreductase. PLoS One.

[29] Raina J-B, Fernandez V, Lambert B, Stocker R, Seymour JR (2019). The role of microbial motility and chemotaxis in symbiosis. Nat Rev Microbiol.

[30] Kelly MS, Barker MF, McKenzie JD, Powell J (1995). The incidence and morphology of subcuticular bacteria in the echinoderm fauna of New Zealand. Biol Bull.

[31] Jackson EW, Pepe-Ranney C, Debenport SJ, Buckley DH, Hewson I (2018). The microbial landscape of sea stars and the anatomical and interspecies variability of their microbiome. Front Microbiol.

[32] Wahl M, Goecke F, Labes A, Dobretsov S, Weinberger F. The second skin: ecological role of epibiotic biofilms on marine organisms. Front Microbiol. 2012;3. 10.3389/fmicb.2012.00292 PMC3425911 22936927

[33] Walker CW, Lesser MP (1989). Nutrition and development of brooded embryos in the brittlestar Amphipholis squamata: do endosymbiotic bacteria play a role?. Mar Biol.

[34] Strahl ED, Dobson WE, Lundie LL (2002). Isolation and screening of brittlestar-associated bacteria for antibacterial activity. Curr Microbiol.

[35] Haszprunar G, Spies M (2014). An integrative approach to the taxonomy of the crown-of-thorns starfish species group (*Asteroidea: Acanthaster*): a review of names and comparison to recent molecular data. Zootaxa..

[36] Yasuda N, Taquet C, Nagai S, Yoshida T, Adjeroud M (2015). Genetic connectivity of the coral-eating sea star *Acanthaster planci* during the severe outbreak of 2006–2009 in the Society Islands, French Polynesia. Mar Ecol.

[37] Apprill A, McNally S, Parsons R, Weber L (2015). Minor revision to V4 region SSU rRNA 806R gene primer greatly increases detection of SAR11 bacterioplankton. Aquat Microb Ecol.

[38] Walters W, Hyde ER, Berg-Lyons D, Ackermann G, Humphrey G, Parada A (2016). Improved bacterial 16S rRNA gene (V4 and V4-5) and fungal internal transcribed spacer marker gene primers for microbial community surveys. mSystems.

[39] Edgar RC (2010). Search and clustering orders of magnitude faster than BLAST. Bioinformatics..

[40] Schloss PD, Westcott SL, Ryabin T, Hall JR, Hartmann M, Hollister EB (2009). Introducing mothur: open-source, platform-independent, community-supported software for describing and comparing microbial communities. Appl Environ Microbiol.

[41] Wada N, Pollock FJ, Willis BL, Ainsworth T, Mano N, Bourne DG (2016). In situ visualization of bacterial populations in coral tissues: pitfalls and solutions. PeerJ..

[42] Daims H, Brühl A, Amann R, Schleifer KH, Wagner M (1999). The domain-specific probe EUB338 is insufficient for the detection of all Bacteria: development and evaluation of a more comprehensive probe set. Syst Appl Microbiol.

[43] Wallner G, Amann R, Beisker W (1993). Optimizing fluorescent in situ hybridization with rRNA-targeted oligonucleotide probes for flow cytometric identification of microorganisms. Cytometry..

[44] Kajitani R, Toshimoto K, Noguchi H, Toyoda A, Ogura Y, Okuno M (2014). Efficient de novo assembly of highly heterozygous genomes from whole-genome shotgun short reads. Genome Res.

[45] Kajitani R, Yoshimura D, Okuno M, Minakuchi Y, Kagoshima H, Fujiyama A (2019). Platanus-allee is a de novo haplotype assembler enabling a comprehensive access to divergent heterozygous regions. Nat Commun.

[46] Stothard P, Wishart DS (2005). Circular genome visualization and exploration using CGView. Bioinformatics..

[47] Seemann T (2014). Prokka: rapid prokaryotic genome annotation. Bioinformatics..

[48] Kanehisa M, Sato Y, Morishima K (2016). BlastKOALA and GhostKOALA: KEGG tools for functional characterization of genome and metagenome sequences. J Mol Biol.

[49] Aramaki T, Blanc-Mathieu R, Endo H, Ohkubo K, Kanehisa M, Goto S, et al. KofamKOALA: KEGG ortholog assignment based on profile HMM and adaptive score threshold. Bioinformatics. 2019;btz859. 10.1093/bioinformatics/btz859 PMC7141845 31742321

