# Supplementary material for: A ubiquitous subcuticular bacterial symbiont of a coral predator, the crown-of-thorns starfish, in the Indo-Pacific
Source: Microbiome. 2020 Aug 24;8:123. doi: 10.1186/s40168-020-00880-3 (PMC7444263; doi:10.1186/s40168-020-00880-3)
Supplement: Supplementary file 2 — Additional file 1: Suppl. Table S1. Sample number of body parts from COTS in the 16S rRNA metabarcoding analysis (total 130 samples) [file 40168_2020_880_MOESM1_ESM.pdf]

Supplementary Table S1

A ubiquitous subcuticular bacterial symbiont of a coral predator, the crown-of-thorns starfish, in the Indo-Pacific

Naohisa WADA, Hideaki YUASA, Rei KAJITANI, Yasuhiro GOTOH, Yoshitoshi OGURA, Dai YOSHIMURA, Atsushi TOYODA, Sen-Lin TANG, Yukio HIGASHIMURA, Hugh SWEATMAN, Zac FORSMAN, Omri BRONSTEIN, Gal EYAL, Nalinee THONGTHAM, Takehiko ITOH, Tetsuya HAYASHI, Nina YASUDA

Suppl. Table S1 Sample number of body parts from COTS in the 16S rRNA metabarcoding analysis (total 130 samples)

	Okinawa			Miyazaki		
	**Okinawa1**	**Okinawa2**	**Okinawa3**	**Miyazaki1**	**Miyazaki2**	**Miyazaki3**
**Surface body parts**						
**Aboral side**						
**Disc spines**						
**Tips**	3	3	3	3	3	3
**Bases**	3	3	3	2	3	3
**Arm spines**						
**Tips**	3	3	3	3	2	3
**Bases**	3	3	3	3	3	3
**Oral side**						
Ambulacral spines						
**Tips**	3	3	3	–	–	–
**Bases**	3	3	3	–	–	–
**Whole**	–	–	–	2	2	2
**Tube foot**	3	3	3	3	3	3
**Internal body parts**						
**Pyloric stomachs**	3	3	3	3	3	3
**Total**	24	24	24	19	19	20
