# Supplementary material for: A ubiquitous subcuticular bacterial symbiont of a coral predator, the crown-of-thorns starfish, in the Indo-Pacific
Source: Microbiome. 2020 Aug 24;8:123. doi: 10.1186/s40168-020-00880-3 (PMC7444263; doi:10.1186/s40168-020-00880-3)
Supplement: Supplementary file 3 — Additional file 2: Suppl. Table S2. Number of OTUs and sequence reads obtained from the 16S rRNA metabarcoding [file 40168_2020_880_MOESM2_ESM.pdf]

Supplementary Table 2

A ubiquitous subcuticular bacterial symbiont of a coral predator, the crown-of-thorns starfish, in the Indo-Pacific

Naohisa WADA, Hideaki YUASA, Rei KAJITANI, Yasuhiro GOTOH, Yoshitoshi OGURA, Dai YOSHIMURA, Atsushi TOYODA, Sen-Lin TANG, Yukio HIGASHIMURA, Hugh SWEATMAN, Zac FORSMAN, Omri BRONSTEIN, Gal EYAL, Nalinee THONGTHAM, Takehiko ITOH, Tetsuya HAYASHI, Nina YASUDA

Supp. table S2 Number of OTUs and sequence reads obtained from the 16S rRNA metabarcoding

Sample ID	Seq. ID	Total		Archaea		Eukaryota		Chloroplast		Unknown		Bacteria		
		OTUs	Reads	OTUs	Reads	OTUs	Reads	OTUs	Reads	OTUs	Reads	OTUs	Reads	OTU1
Okinawa1_Disk Spine tip 1	O1Dt1	67	17783	0	0	37	1158	0	0	4	1951	26	14674	10217
Okinawa1_Disk Spine tip 2	O1Dt2	146	15633	0	0	18	310	8	250	6	425	114	14648	2354
Okinawa1_Disk Spine tip 3	O1Dt3	72	21542	0	0	23	1018	0	0	7	1584	42	18940	7265
Okinawa2_Disk Spine tip 1	O2Dt1	19	23861	0	0	0	0	0	0	0	0	19	23861	0
Okinawa2_Disk Spine tip 2	O2Dt2	94	11125	0	0	18	39	5	297	4	563	67	10226	4723
Okinawa2_Disk Spine tip 3	O2Dt3	63	16127	0	0	32	106	0	0	4	1609	27	14412	12027
Okinawa3_Disk Spine tip 1	O3Dt1	84	11569	0	0	18	51	1	10	4	667	61	10841	7154
Okinawa3_Disk Spine tip 2	O3Dt2	114	13252	0	0	16	36	4	26	4	708	90	12482	3301
Okinawa3_Disk Spine tip 3	O3Dt3	72	16091	0	0	14	63	0	0	5	2564	53	13464	10000
Okinawa1_Disk Spine base 1	O1Db1	89	17899	0	0	53	2237	0	0	7	3184	29	12478	11470
Okinawa1_Disk Spine base 2	O1Db2	85	17065	0	0	53	2132	0	0	4	2802	28	12131	10835
Okinawa1_Disk Spine base 3	O1Db3	83	17203	0	0	52	2115	0	0	7	3173	24	11915	10734
Okinawa2_Disk Spine base 1	O2Db1	32	8870	0	0	8	20	0	0	3	628	21	8222	6920
Okinawa2_Disk Spine base 2	O2Db2	37	15995	0	0	10	23	0	0	3	1212	24	14760	11031
Okinawa2_Disk Spine base 3	O2Db3	17	17591	0	0	1	376	0	0	0	0	16	17215	0
Okinawa3_Disk Spine base 1	O3Db1	37	22515	0	0	3	12	0	0	4	5729	30	16774	10924
Okinawa3_Disk Spine base 2	O3Db2	47	23933	0	0	12	36	0	0	4	3252	31	20645	16772
Okinawa3_Disk Spine base 3	O3Db3	65	15928	0	0	26	150	0	0	7	1461	32	14317	11485
Miyazaki1_Disk Spine tip 1	M1Dt1	69	16664	0	0	31	196	0	0	5	5783	33	10685	6217

Miyazaki1_Disk Spine tip 2	M1Dt2	79	11535	0	0	36	148	0	0	9	5122	34	6265	4734
Miyazaki1_Disk Spine tip 3	M1Dt3	90	18574	0	0	26	169	16	786	6	9149	42	8470	3769
Miyazaki2_Disk Spine tip 1	M2Dt1	111	14465	0	0	13	31	16	1708	7	2621	75	10105	7699
Miyazaki2_Disk Spine tip 2	M2Dt2	63	14781	2	20	17	29	2	19	3	1991	39	12722	11338
Miyazaki2_Disk Spine tip 3	M2Dt3	54	16897	1	2	1	4	2	62	3	2058	47	14771	12064
Miyazaki3_Disk Spine tip 1	M3Dt1	42	14456	0	0	15	28	0	0	3	1478	24	12950	11823
Miyazaki3_Disk Spine tip 2	M3Dt2	57	15567	0	0	16	24	3	10	3	1229	35	14304	13683
Miyazaki3_Disk Spine tip 3	M3Dt3	76	18549	0	0	24	108	6	50	6	2931	40	15460	14108
Miyazaki1_Disk Spine base 2	M1Db2	33	17864	0	0	2	28	0	0	3	5155	28	12681	5003
Miyazaki1_Disk Spine base 3	M1Db3	34	23321	0	0	1	10	2	80	3	5872	28	17359	7552
Miyazaki2_Disk Spine base 1	M2Db1	38	18732	1	5	1	3	2	10	3	2478	31	16236	11125
Miyazaki2_Disk Spine base 2	M2Db2	42	12599	0	0	8	15	0	0	3	2646	31	9938	8823
Miyazaki2_Disk Spine base 3	M2Db3	38	16239	0	0	2	3	1	1	3	1800	32	14435	10015
Miyazaki3_Disk Spine base 1	M3Db1	44	15517	0	0	2	8	4	43	3	1109	35	14357	10086
Miyazaki3_Disk Spine base 2	M3Db2	38	15143	0	0	7	24	0	0	3	1491	28	13628	10855
Miyazaki3_Disk Spine base 3	M3Db3	34	16885	0	0	7	30	0	0	4	1740	23	15115	12990
Okinawa1_Arm Spine tip 1	O1At1	60	17906	0	0	32	1336	0	0	3	2093	25	14477	12028
Okinawa1_Arm Spine tip 2	O1At2	56	13628	0	0	38	1436	0	0	5	2221	13	9971	9203
Okinawa1_Arm Spine tip 3	O1At3	44	17483	0	0	24	1199	0	0	2	1853	18	14431	8699
Okinawa2_Arm Spine tip 1	O2At1	101	16407	0	0	32	125	3	49	4	2343	62	13890	6023
Okinawa2_Arm Spine tip 2	O2At2	63	12766	0	0	32	100	0	0	5	1801	26	10865	9339
Okinawa2_Arm Spine tip 3	O2At3	68	20942	0	0	34	173	0	0	6	3823	28	16946	14171

Okinawa3_Arm Spine tip 1	O3At1	78	14624	0	0	26	98	0	0	5	2367	47	12159	10185
Okinawa3_Arm Spine tip 2	O3At2	25	11307	0	0	2	70	0	0	4	24	19	11213	0
Okinawa3_Arm Spine tip 3	O3At3	56	17211	0	0	20	95	0	0	5	2964	31	14152	11949
Okinawa1_Arm Spine base 1	O1Ab1	60	16033	0	0	33	1533	0	0	3	2239	24	12261	10933
Okinawa1_Arm Spine base 2	O1Ab2	76	16995	0	0	48	2350	0	0	8	3591	20	11054	10025
Okinawa1_Arm Spine base 3	O1Ab3	53	17307	0	0	30	911	0	0	5	1233	18	15163	14136
Okinawa2_Arm Spine base 1	O2Ab1	65	16333	0	0	30	120	0	0	3	1404	32	14809	11674
Okinawa2_Arm Spine base 2	O2Ab2	55	19710	0	0	15	45	0	0	6	1119	34	18546	15875
Okinawa2_Arm Spine base 3	O2Ab3	45	17408	0	0	18	32	0	0	4	1157	23	16219	5834
Okinawa3_Arm Spine base 1	O3Ab1	45	12844	0	0	6	12	0	0	5	1430	34	11402	6866
Okinawa3_Arm Spine base 2	O3Ab2	30	14578	0	0	5	9	0	0	4	2044	21	12525	8809
Okinawa3_Arm Spine base 3	O3Ab3	31	14994	0	0	7	88	0	0	4	2321	20	12585	9798
Miyazaki1_Arm Spine tip 1	M1At1	88	15614	1	31	42	235	5	74	6	4567	34	10707	6501
Miyazaki1_Arm Spine tip 2	M1At2	92	14205	0	0	48	295	2	14	9	6238	33	7658	5298
Miyazaki1_Arm Spine tip 3	M1At3	54	18626	0	0	17	111	2	110	6	5163	29	13242	10516
Miyazaki2_Arm Spine tip 1	M2At1	118	15769	7	80	1	2	18	823	4	1689	88	13175	9326
Miyazaki2_Arm Spine tip 3	M2At3	57	16220	0	0	23	53	0	0	7	2665	27	13502	12932
Miyazaki3_Arm Spine tip 1	M3At1	66	16590	0	0	26	72	2	3	4	1599	34	14916	11060
Miyazaki3_Arm Spine tip 2	M3At2	73	13477	0	0	35	85	4	10	4	1888	30	11494	8937
Miyazaki3_Arm Spine tip 3	M3At3	60	16339	0	0	23	72	0	0	6	2459	31	13808	11239
Miyazaki1_Arm Spine base 1	M1Ab1	35	16398	0	0	5	42	0	0	4	5281	26	11075	5266
Miyazaki1_Arm Spine base 2	M1Ab2	44	14705	0	0	5	51	3	12	3	3013	33	11629	7010

Miyazaki1_Arm Spine base 3	M1Ab3	31	18591	0	0	3	7	0	0	4	3071	24	15513	3604
Miyazaki2_Arm Spine base 1	M2Ab1	36	14550	0	0	2	6	0	0	3	3421	31	11123	9826
Miyazaki2_Arm Spine base 2	M2Ab2	27	12266	0	0	1	1	1	5	3	2484	22	9776	8994
Miyazaki2_Arm Spine base 3	M2Ab3	39	14460	1	2	1	1	4	8	3	1675	30	12774	11399
Miyazaki3_Arm Spine base 1	M3Ab1	21	15976	0	0	0	0	0	0	1	1	20	15975	1
Miyazaki3_Arm Spine base 2	M3Ab2	35	19258	0	0	1	55	0	0	3	10198	31	9005	123
Miyazaki3_Arm Spine base 3	M3Ab3	72	19177	2	16	5	29	3	34	3	2295	59	16803	11694
Okinawa1_Ambulacral Spine tip1	O1Bt1	83	17618	1	40	29	571	4	155	5	667	44	16185	10721
Okinawa1_Ambulacral Spine tip2	O1Bt2	47	12528	0	0	19	852	0	0	5	1218	23	10458	7529
Okinawa1_Ambulacral Spine tip3	O1Bt3	72	17616	0	0	37	2210	3	109	8	3097	24	12200	8313
Okinawa2_Ambulacral Spine tip 1	O2Bt1	54	15746	0	0	21	88	0	0	5	2470	28	13188	6848
Okinawa2_Ambulacral Spine tip 2	O2Bt2	92	12408	0	0	18	64	7	68	4	715	63	11561	6302
Okinawa2_Ambulacral Spine tip 3	O2Bt3	61	16696	0	0	29	120	0	0	7	2262	25	14314	7911
Okinawa3_Ambulacral Spine tip 1	O3Bt1	115	12372	0	0	42	236	1	6	7	3458	65	8672	5184
Okinawa3_Ambulacral Spine tip 2	O3Bt2	72	9325	0	0	37	124	1	2	5	1703	29	7496	6475
Okinawa3_Ambulacral Spine tip 3	O3Bt3	100	13316	0	0	43	241	0	0	7	2857	50	10218	8401
Okinawa1_Ambulacral Spine base 1	O1Bb1	62	15033	0	0	44	969	0	0	2	1273	16	12791	11322
Okinawa1_Ambulacral Spine base 2	O1Bb2	58	17913	0	0	32	1112	0	0	2	1562	24	15239	11836
Okinawa1_Ambulacral Spine base 3	O1Bb3	67	16846	0	0	37	1058	0	0	3	1400	27	14388	11108
Okinawa2_Ambulacral Spine base 1	O2Bb1	61	18228	0	0	28	115	2	10	4	1167	27	16936	11036
Okinawa2_Ambulacral Spine base 2	O2Bb2	51	19758	0	0	17	51	0	0	4	1746	30	17961	12001
Okinawa2_Ambulacral Spine base 3	O2Bb3	53	21947	0	0	23	118	0	0	4	2267	26	19562	11438

Okinawa3_Ambulacral Spine base 1	O3Bb1	54	2592	0	0	24	79	0	0	5	1019	25	1494	579
Okinawa3_Ambulacral Spine base 2	O3Bb2	58	11467	0	0	34	121	0	0	3	1929	21	9417	7789
Okinawa3_Ambulacral Spine base 3	O3Bb3	74	11681	0	0	37	183	0	0	7	3247	30	8251	5404
Miyazaki1_Ambulacral Spine 1	M1Bw1	41	15418	1	13	3	9	1	1	3	2879	33	12516	11871
Miyazaki1_Ambulacral Spine 2	M1Bw2	59	14110	0	0	25	125	1	11	7	6033	26	7941	7450
Miyazaki2_Ambulacral Spine 1	M2Bw1	46	13053	0	0	14	19	0	0	3	2670	29	10364	9950
Miyazaki2_Ambulacral Spine 2	M2Bw2	62	14415	1	7	32	85	1	2	4	3123	24	11198	10729
Miyazaki3_Ambulacral Spine 1	M3Bw1	79	11965	1	16	30	93	5	30	4	2384	39	9442	7657
Miyazaki3_Ambulacral Spine 2	M3Bw2	71	15123	0	0	35	98	0	0	6	3209	30	11816	10272
Okinawa1_Tube feet 1	O1T1	75	9470	0	0	54	1701	0	0	4	2392	17	5377	5175
Okinawa1_Tube feet 2	O1T2	74	9160	0	0	49	1403	0	0	4	2045	21	5712	4693
Okinawa1_Tube feet 3	O1T3	65	11697	0	0	42	991	0	0	3	1362	20	9344	9044
Okinawa2_Tube feet 1	O2T1	82	14484	0	0	43	188	0	0	9	2066	30	12230	9646
Okinawa2_Tube feet 2	O2T2	69	16941	0	0	15	35	0	0	5	243	49	16663	1362
Okinawa2_Tube feet 3	O2T3	96	13831	0	0	59	467	0	0	10	4214	27	9150	6024
Okinawa3_Tube feet 1	O3T1	97	12706	0	0	45	320	0	0	6	5404	46	6982	4753
Okinawa3_Tube feet 2	O3T2	78	9280	0	0	47	345	0	0	7	2498	24	6437	5609
Okinawa3_Tube feet 3	O3T3	99	10321	0	0	65	592	1	20	8	4540	25	5169	3992
Miyazaki1_Tube feet 1	M1T1	71	15623	0	0	27	130	0	0	7	4952	37	10541	9057
Miyazaki1_Tube feet 2	M1T2	56	15101	0	0	19	72	1	1	6	3345	30	11683	9770
Miyazaki1_Tube feet 3	M1T3	45	16813	0	0	10	69	0	0	4	3418	31	13326	10008
Miyazaki2_Tube feet 1	M2T1	109	16685	0	0	62	642	0	0	13	10012	34	6031	5146

Miyazaki2_Tube feet 2	M2T2	91	7365	0	0	58	331	0	0	8	4434	25	2600	2391
Miyazaki2_Tube feet 3	M2T3	100	13669	0	0	63	369	0	0	10	6649	27	6651	5977
Miyazaki3_Tube feet 1	M3T1	71	11791	0	0	41	108	0	0	4	2136	26	9547	8597
Miyazaki3_Tube feet 2	M3T2	59	12925	0	0	23	62	0	0	4	1826	32	11037	9799
Miyazaki3_Tube feet 3	M3T3	47	14942	0	0	12	20	0	0	3	2415	32	12507	9043
Okinawa1_Stomach 1	O1S1	96	7968	0	0	69	3146	0	0	6	4099	21	723	12
Okinawa1_Stomach 2	O1S2	105	7511	0	0	71	3031	0	0	9	3777	25	703	1
Okinawa1_Stomach 3	O1S3	37	16731	0	0	9	1270	0	0	3	1903	25	13558	1
Okinawa2_Stomach 1	O2S1	107	7061	0	0	75	688	0	0	10	5860	22	513	59
Okinawa2_Stomach 2	O2S2	92	7927	0	0	62	597	0	0	7	5162	23	2168	1306
Okinawa2_Stomach 3	O2S3	108	11561	0	0	78	788	0	0	8	9540	22	1233	146
Okinawa3_Stomach 1	O3S1	100	9142	0	0	73	795	0	0	9	5506	18	2841	1
Okinawa3_Stomach 2	O3S2	97	10874	0	0	70	527	0	0	8	3841	19	6506	1
Okinawa3_Stomach 3	O3S3	103	9945	0	0	73	766	1	10	7	7519	22	1650	10
Miyazaki1_Stomach 1	M1S1	114	15446	0	0	60	843	1	1	12	13128	41	1474	148
Miyazaki1_Stomach 2	M1S2	139	14178	4	22	53	738	15	125	8	12258	59	1035	53
Miyazaki1_Stomach 3	M1S3	104	19969	0	0	51	447	0	0	9	15890	44	3632	30
Miyazaki2_Stomach 1	M2S1	107	14303	0	0	72	686	0	0	10	12573	25	1044	73
Miyazaki2_Stomach 2	M2S2	140	15132	0	0	81	829	4	10	12	13677	43	616	25
Miyazaki2_Stomach 3	M2S3	129	20375	1	7	64	570	6	67	8	18547	50	1184	27
Miyazaki3_Stomach 1	M3S1	99	14411	2	3	32	526	2	3	6	11820	57	2059	276
Miyazaki3_Stomach 2	M3S2	65	14795	1	2	19	448	1	1	5	11626	39	2718	353

Miyazaki3_Stomach 3	M3S3	109	25351	0	0	43	507	0	0	6	21855	60	2989	77
Seawater_Okinawa1	OSW1	255	18533	12	667	0	0	29	1187	1	1	213	16678	5
Seawater_Okinawa2	OSW2	268	23260	10	954	0	0	29	1446	1	1	228	20859	6
Seawater_Okinawa3	OSW3	282	23807	12	674	0	0	30	1473	1	1	239	21659	4
Seawater_Miyazaki1	MSW1	343	19960	26	2551	0	0	40	2218	1	1	276	15190	0
Seawater_Miyazaki2	MSW2	355	21131	26	3370	0	0	43	3074	2	3	284	14684	0
Seawater_Miyazaki3	MSW3	352	28661	26	4204	0	0	43	5191	1	2	282	19264	0
