# Supplementary material for: A ubiquitous subcuticular bacterial symbiont of a coral predator, the crown-of-thorns starfish, in the Indo-Pacific
Source: Microbiome. 2020 Aug 24;8:123. doi: 10.1186/s40168-020-00880-3 (PMC7444263; doi:10.1186/s40168-020-00880-3)
Supplement: Supplementary file 4 — Additional file 3: Suppl. Table S3. Bacterial taxa number identified from phylum to family in the COTSs and seawater samples (total 761 OTUs). Suppl. Table S4. Abundance of COTS27 (OTU1) in the different body components of COTS among Okinawa and Miyazaki samples. Suppl. Table S5. COTS genome sequencing read data. All reads were preprocessed to exclude adaptor sequences and low-quality bases by Platanus_trim (version 1.0.7; http://platanus.bio.titech.ac.jp/). For all mate-pair libraries, short-insert pairs (estimated insert size ≤ 0.5 × nominal size) and PCR products, duplicates were removed based on mapping information to the assembled results (scaffolds), which were constructed only from the paired-end libraries, using an in-house program. Suppl. Table S6. COTS27 genome information. Suppl. Table S7. COTS27 genome general biosynthesis profile based on KEGG metabolic pathways. Suppl. Table S8. Samples used for 16S rRNA metabarcoding, reconstruction of phylogenetic tree, and PCR screening and sequencing. Suppl. Table S9. Primers used for 16S rRNA gene sequence analysis and probes for FISH used in this study [file 40168_2020_880_MOESM3_ESM.pdf]

Supplementary Tables S3-9

A ubiquitous subcuticular bacterial symbiont of a coral predator, the crown-of-thorns starfish, in the Indo-Pacific

Naohisa WADA, Hideaki YUASA, Rei KAJITANI, Yasuhiro GOTOH, Yoshitoshi OGURA, Dai YOSHIMURA, Atsushi TOYODA, Sen-Lin TANG, Yukio HIGASHIMURA, Hugh SWEATMAN, Zac FORSMAN, Omri BRONSTEIN, Gal EYAL, Nalinee THONGTHAM, Takehiko ITOH, Tetsuya HAYASHI, Nina YASUDA

Suppl. table S3 Bacterial taxa number identified from phylum to family in the COTSs and seawater samples (total 761 OTUs).

	COTS			Seawater		
	Total	Okinawan individuals (n=3)	Miyazaki's individuals (n=3)	Total	Okinawan seawater (n=3)	Miyazaki's seawater (n=3)
Phylum	19	15	17	16	15	15
Class	34 (1)	26	28 (1)	24 (1)	21 (1)	21 (1)
Order	92 (6)	68 (5)	73 (4)	68 (5)	57 (5)	62 (4)
Family	144 (5)	109 (1)	110 (5)	96 (3)	79 (2)	89 (3)
Unclassified phylum	29 OTUs	20 OTUs	17 OTUs	22 OTUs	17 OTUs	17 OTUs
Unknown kingdom *1	7 OTUs	3 OTUs	5 OTUs	12 OTUs	11 OTUs	10 OTUs

The number of bacterial taxa and their corresponding taxonomic level. The number of both unclassified or unknown bacterial taxa are mentioned in brackets.

***1 The OTUs were classified as bacteria in the Silva SINA aligner (https://www.arb-silva.de/aligner/) after sorting in MOTHUR (https://mothur.org/).**

Suppl. table S4 Abundance of COTS27 (OTU1) in the different body components of COTS among Okinawa and Miyazaki samples

	**Average abundance of COTS27 (OTU1) (%)**		
	**Total**	**Okinawa**	**Miyazaki**
**Surface body parts**			
**All spines**	**68.8 ± 24.1 (n=94)**	**66.5 ± 24.1 (n=54)**	**72.0 ± 24.1 (n=40)**
Aboral side			
Disc spines	66.5 ± 26.0 (n=35)	59.8 ± 30.9 (n=18)	73.5 ± 17.8 (n=17)
Tips	62.4 ± 28.1 (n=18)	46.7 ± 28.7 (n=9)	78.2 ± 17.0 (n=9)
Bases	70.7 ± 23.7 (n=17)	73.0 ± 28.6 (n=9)	68.2 ± 18.2 (n=8)
Arm spines	68.0 ± 26.9 (n=35)	72.1 ± 24.4 (n=18)	63.6 ± 29.5 (n=17)
Tips	72.1 ± 22.6 (n=17)	68.5 ± 30.0 (n=9)	76.1 ± 10.3 (n=8)
Bases	64.1 ± 30.6 (n=18)	75.8 ± 18.3 (n=9)	52.4 ± 36.7 (n=9)
Oral side			
Ambulacral spines	73.6 ± 15.8 (n=24)	67.6 ± 13.3 (n=18)	91.4 ± 6.1 (n=6)
Tips	66.3 ± 12.3 (n=9)	66.3 ± 12.3 (n=9)	–
Bases	69.0 ± 14.9 (n=9)	69.0 ± 14.9 (n=9)	–
Whole	91.4 ± 6.1 (n=6)	–	91.4 ± 6.1 (n=6)
**Tube feet**	**79.1 ± 19.8 (n=18)**	**73.4 ± 26.7 (n=9)**	**84.8 ± 6.8 (n=9)**
**Internal body parts**			
**Pyloric stomachs**	**8.0 ± 13.9 (n=18)**	**9.6 ± 19.6 (n=9)**	**6.5 ± 4.7 (n=9)**

Suppl. table S5 COTS genome sequencing read data. All reads were preprocessed to exclude adaptor sequences and low-quality bases by Platanus_trim (version 1.0.7; http://platanus.bio.titech.ac.jp/). For all mate-pair libraries, short-insert pairs (estimated insert size ≤ 0.5 × nominal size) and PCR products, duplicates were removed based on mapping information to the assembled results (scaffolds), which were constructed only from the paired-end libraries, using an in-house program.

		Raw		Pre-processed	
Library type	Nominal insert length (bp)	Mean read length (bp)	Total length (bp)	Mean read length (bp)	Total length (bp)
paired-ends	300	150	33,596,575,800	147	32,610,796,353
paired-ends	500	150	34,403,911,800	147	32,812,079,166
mate-pairs	3,000	150	21,372,289,200	116	11,201,114,333
mate-pairs	5,000	150	22,326,121,800	118	11,998,456,502
mate-pairs	8,000	150	22,750,867,500	120	12,396,355,042
mate-pairs	10,000	150	22,390,767,000	121	11,476,430,100
mate-pairs	12,000	150	22,224,980,700	119	10,842,998,778
mate-pairs	15,000	150	24,490,445,400	120	11,070,146,690

Suppl. table S6 COTS27 genome information

COTS27 genome	
Genome size	2,684,921 bp
Gaps	23
Total number of Ns	392 bp
GC ratio	39.59%
Proteins	1,650
tRNAs	35
rRNAs	3

Suppl. table S7 COTS27 genome general biosynthesis profile based on KEGG metabolic pathways

Biosynthesis	States*1	Biosynthesis	States*1
Amino acid biosynthesis		***Metabolism of cofactors and vitamins***	
Alanine	+ +	Thiamine	–
Arginine	+ +	Riboflavin *2	+ +
Asparagine	–	Pyridoxal	+
Aspartic acid	–	NAD	+ +
Cysteine	+ +	Pantothenate	–
Glutamine	+ +	Coenzyme A	+ +
Glutamic acid	+ +	Pimeloyl-ACP	–
Glycine	+	Biotin	+ +
Histidine	+	Tetrahydrofolate	+
Isoleucine	+ +	L-threo-Tetrahydrobiopterin	–
Leucine	+ +	C1-unit interconversion	+ +
Lysine	+ +	Hemo	–
Methionine	+ +	Siroheme	–
Phenylalanine	+ +	Menaquinone	–
Proline	+ +	***Lipid metabolism***	
Serine	+	***•Fatty acid biosynthesis degradation***	
Threonine	+	Fatty acid	+ +
Tryptophan	+ +	Beta-Oxidation	+
Tyrosine	+ +	***•Lipid metabolism***	
Valine	+ +	Phosphatidylethanolamine (PE)	+ +
Nucleotide biosynthesis		Ketone body	–
•Purine		Triacylglycerol	–
Inosine monophosphate	+ +		
Adenine ribonucleotide	+ +		
Guanine ribonucleotide	+		
•Pyrimidine			
Uridine monophosphate	+ +		
Pyrimidine ribonucleotide	+ +		
Pyrimidine deoxy ribonucleotide	+ +		

*1: Indication of the biosynthesis pathway prediction: complete (+ +), one block missing (+), and two or more blocks missing (–).

*2: Not including biosynthesis pathway of flavin adenine dinucleotide (FAD) and flavin mononucleotide (FMN).

Suppl. table S8 Samples used for 16S rRNA metabarcoding, reconstruction of phylogenetic tree, and PCR screening and sequencing

Analysis	Location I.D.	*n* [*1]	Location	Country	Year	Remarks
1. 16S rRNA metabarcoding						
	Miyazaki	3	Ohshima, Miyazaki	Japan	Nov. 2017	This study
	Okinawa	3	Yamakawa, Okinawa	Japan	Jul. 2017	This study
2. Phylogenetic analysis of COTS27						
	Miyazaki	3	Ohshima, Miyazaki	Japan	Nov. 2017	Same specimens with 1.
	Okinawa	2	Yamakawa, Okinawa	Japan	Jul. 2017	Same specimens with 1.
3. PCR screening and sequencing of COTS27						
	Wakayama	16	Wakayama	Japan	Dec. 2005–Jun. 2006	[1]
	Tatsukushi	6	Kouchi	Japan	Nov. 2005	[1]
	Sakura-jima	9	Kagoshima	Japan	2004–2005	[1]
	Amami-Ohshima	6	Kagoshima	Japan	May–Sep. 2004	[1]
	Onna-village	18	Okinawa	Japan	Jun. 2013	This study
	Kerama Island	9	Okinawa	Japan	Jul. 2004	[2]
	Kume Island	16	Okinawa	Japan	Sep. 2005	[1]
	Miyako Island	6	Okinawa	Japan	May–Sep. 2004	[1]
	Sekisei Lagoon,	12	Okinawa	Japan	2004	This study
	Bowden Reef	16	Great Barrier Reef	Australia	Mar. 2007	[1]
	Clack Reef	18	Great Barrier Reef	Australia	Nov. 2006	[1]
	Shell Reef	28	Great Barrier Reef	Australia	Mar. 2007	[1]

	Hawaii	23	Hawaii	United States	2007–Sep. 2014	This study
	Phuket	4	Phuket	Thailand	Oct. 2017–Aug. 2017	This study
	Eilat	8	Eilat	Israel	Feb. 2017	This study
4. Fluorescence *in situ* hybridization (FISH)						
	Miyazaki	3	Ohshima, Miyazaki	Japan	Apr. 2017	This study
5. Hologenome sequencing analysis						
	Miyazaki	1	Ohshima, Miyazaki	Japan	Aug. 2014	This study

*1 *n* = number of the individuals

References;

1. Yasuda N, Nagai S, Hamaguchi M, Okaji K, Gérard K, Nadaoka K. Gene flow of Acanthaster planci (L.) in relation to ocean currents revealed by microsatellite analysis. *Mol Ecol* 2009; **18**: 1574–1590.

2. Yasuda N, Ogasawara K, Kajiwara K, Ueno M, Oki K, Taniguchi H, et al. Latitudinal differentiation in the reproduction patterns of the crown-of-thorns starfish Acanthaster planci through the Ryukyu Island Archipelago. *Plankton Benthos Res* 2010; **5**: 156–164.

Suppl. table S9 Primers used for 16S rRNA gene sequence analysis and probes for FISH used in this study

Analysis	Name	Sequence (5 '–3 ')	Targeted organism	*E. coli* position	Ref. and remarks
16S rRNA metabarcoding	515F_EMP	TCGTCGGCAGCGTCAGATGTGTATAAGAGACAG-GTGYCAGCMGCCGCGGTAA	Most bacteria	515	[1, 2]
	806rb_EMP	GTCTCGTGGGCTCGGAGATGTGTATAAGAGACAG-GGACTACNVGGGTWTCTAAT	Most bacteria	806	[1, 2]
Phylogenetic analysis of COTS27 using the full-length 16S rRNA gene sequence	27F	AGAGTTTGATCMTGGCTCAG	Most bacteria	27	[3]
	COTS_V4_R	CCTACACCAGGAATTCCGACTA	COTS27	668	This study
	COTS_V4R_F	TAGTCGGAATTCCTGGTGTAGG	COTS27	668	This study
	1492R(c)	TACGGTTACCTTGTTACGAC	Most bacteria	1492	[4]
	Microbiont F	GAGCAATCTCACATGGATGACG	COTS27	392	This study
	Microbiont R	CATGCTGATCCGCGATTACTAG	COTS27	1342	This study
PCR screening and sequencing of COTS27	COTSsymb_F	GATAGCCGCTGTAATGGCGA	COTS27	148	This study
	COTSsymb_R	AGGCCTTCGTCATCCATGTG	COTS27	399	This study
	Hitode_16S_f*3	TGACYGTGCRAAGGTAGRATAATCATTGC	*Asteroidea*-universal primer	–	This study
	Hitode_16S_r*3	CGCTGTTATCCCTRYGGSAACTT	*Asteroidea*-universal primer	–	This study
FISH	COTSsymb*1	CTCAGCGATGCTAACGCACC	COTS27	196	This study, FA 30% *2
	EUB338mix*1	GCWGCCWCCCGTAGGWGT	Most bacteria	338	[5, 6], FA 30%

				*2
Non338[1]	ACATCCTACGGGAGGC	–	–	[7], FA 30% *2

*1. Probes included 5'-end fluorescein Cy3 labeled.

*2. Formamide (FA) concentration (v/v) for hybridization at 46˚C.

*3. Primers designed to amplify COTS mitochondrial 16S rRNA gene sequence as a positive control.

References;

1. Apprill A, McNally S, Parsons R, Weber L. Minor revision to V4 region SSU rRNA 806R gene primer greatly increases detection of SAR11 bacterioplankton. *Aquat Microb Ecol* 2015; **75**: 129–137.
2. Walters W, Hyde ER, Berg-Lyons D, Ackermann G, Humphrey G, Parada A, et al. Improved Bacterial 16S rRNA Gene (V4 and V4-5) and Fungal Internal Transcribed Spacer Marker Gene Primers for Microbial Community Surveys. *mSystems* 2016; **1**: e00009-15.
3. Lane D. 16S/23S rRNA sequencing. *Nucleic Acid Tech Bact Syst* 1991; 115–175.
4. Allen MA, Goh F, Burns BP, Neilan BA. Bacterial, archaeal and eukaryotic diversity of smooth and pustular microbial mat communities in the hypersaline lagoon of Shark Bay. *Geobiology* 2009; **7**: 82–96.
5. Daims H, Brühl A, Amann R, Schleifer KH, Wagner M. The domain-specific probe EUB338 is insufficient for the detection of all Bacteria: development and evaluation of a more comprehensive probe set. *Syst Appl Microbiol* 1999; **22**: 434–44.
6. Amann RI, Krumholz L, Stahl DA. Fluorescent-oligonucleotide probing of whole cells for determinative, phylogenetic, and environmental studies in microbiology. *J Bacteriol* 1990; **172**: 762–770.
7. Wallner G, Amann R, Beisker W. Optimizing fluorescent in situ hybridization with rRNA-targeted oligonucleotide probes for flow

cytometric identification of microorganisms. *Cytometry* 1993; **14**: 136–43.
