# Supplementary material for: A ubiquitous subcuticular bacterial symbiont of a coral predator, the crown-of-thorns starfish, in the Indo-Pacific
Source: Microbiome. 2020 Aug 24;8:123. doi: 10.1186/s40168-020-00880-3 (PMC7444263; doi:10.1186/s40168-020-00880-3)
Supplement: Supplementary file 6 — Additional file 5: Appendixes Appendix 1. Other relatively abundant bacteria in COTS. Appendix 2. The members of clade I in marine spirochetes [file 40168_2020_880_MOESM5_ESM.pdf]

APPENDIXES

A ubiquitous subcuticular bacterial symbiont of a coral predator, the crown-of-thorns starfish, in the Indo-Pacific

Naohisa WADA, Hideaki YUASA, Rei KAJITANI, Yasuhiro GOTOH, Yoshitoshi OGURA, Dai YOSHIMURA, Atsushi TOYODA, Sen-Lin TANG, Yukio HIGASHIMURA, Hugh SWEATMAN, Zac FORSMAN, Omri BRONSTEIN, Gal EYAL, Nalinee THONGTHAM, Takehiko ITOH, Tetsuya HAYASHI, Nina YASUDA

Appendix 1 Other relatively abundant bacteria in COTS

Relatively abundant OTUs other than OTU 1 (COTS27) in COTS are shown in Appendix table 1. They belong to the families: *Spiroplasmataceae* (OTU 3, 5.8% average abundance in all COTS samples; OTU 4, 2.4%), *Bacillaceae* (OTU 5, 3.7%; OTU9, 1.9%), *Rhizobiaceae* (OTU 7, 2.8%), *Burkholderiaceae* (OTU 6, 2.4%), *Flavobacteriaceae* (OTU 8, 1.6%), *Francisellaceae* (OTU 11, 1.4%) and *Endozoicomonadaceae* (OTU 10, 0.8%).

Appendix table 1 Other relatively abundant OTUs in COTS

OTU ID, putative taxon	Top BLAST match *1; Accession (% identity)	Average abundance (%) in;			
		Total COTS samples	Spine	Tube feet	Pyloric stomach
OTU 3, *Spiroplasmataieae* *2	MG776020.1 (99%)	5.79	2.83	4.02	23.03
OTU 5, *Bacillaceae*	KF975538.1 (98%)	3.67	3.33	1.19	7.97
OTU 7, *Rhizobiaceae*	JQ387353.2 (99%)	2.77	2.70	0.69	5.24
OTU 6, *Burkholderiaceae*	MG858809.1 (99%)	2.43	2.26	0.70	5.06
OTU 4, *Spiroplasmataieae* *2	MG776020.1 (90%)	2.39	2.54	0.09	3.88
OTU 9, *Bacillaceae*	KY989221.1 (98%)	1.89	1.69	0.49	4.31
OTU 8, *Flavobacteriaceae*	KU578369.1 (92%)	1.62	1.37	2.55	1.98
OTU 11, *Francisellaceae*	FJ202895.1 (98%)	1.35	0.60	0.34	6.23
OTU 10, *Endozoicomonadaceae*	AM495252.1 (98%)	0.77	0.95	0.28	0.31

*1 Top BLAST hits in the NCBI nr/nt database (https://blast.ncbi.nlm.nih.gov/Blast.cgi?PAGE_TYPE=BlastSearch) are shown.

*2 The phylogenies of OTU 3 and OTU 4 were determined in the databases of All-species Living Tree Project and RDP, respectively using Silva SINA [1].

Appendix 2 The members of clade I in marine spirochetes

Among the marine spirochetes, COTS27 formed a distinct clade (clade I in **Fig. 3**) with an uncultured spirochete SRODG048 (GenBank accession No. FM995181) - obtained from Sydney rock oysters in Australia [2], an uncultured spirochete bacterium clone GHI14 (GenBank accession No. EU857763) - detected from crystalline styles of marine bivalves in North sea [3], and an uncultured marine bacterium clone Sp02sw36 (GenBank accession No. HQ241817) - obtained from the sponge *Tsitsikamma favus* in South Africa [4]. However, the 16S rRNA gene of COTS27 shares 85.9–86.9%, 86.4–86.9%, and 84.8–85.4% sequence identity with the three abovementioned clones,

respectively. As these identity values are at the same level as the proposed threshold for assigning a novel bacterial family (86.5%) [5], COTS27 represents a distinct family in clade I.

1. Pruesse E, Peplies J, Glöckner FO. SINA: Accurate high-throughput multiple sequence alignment of ribosomal RNA genes. Bioinformatics. 2012;28:1823–9.

2. Green T j., Barnes A c. Bacterial diversity of the digestive gland of Sydney rock oysters, Saccostrea glomerata infected with the paramyxean parasite, Marteilia sydneyi. J Appl Microbiol. 2010;109:613–22.

3. Husmann G, Gerdts G, Wichels A. Spirochetes in Crystalline Styles of Marine Bivalves: Group-Specific PCR Detection and 16S rRNA Sequence Analysis. J Shellfish Res. 2010;29:1069–75.

4. Walmsley TA, Matcher GF, Zhang F, Hill RT, Davies-Coleman MT, Dorrington RA. Diversity of Bacterial Communities Associated with the Indian Ocean Sponge *Tsitsikamma favus* That Contains the Bioactive Pyrroloiminoquinones, Tsitsikammamine A and B. Mar Biotechnol. 2012;14:681–91.

5. Yarza P, Yilmaz P, Pruesse E, Glöckner FO, Ludwig W, Schleifer K-H, et al. Uniting the classification of cultured and uncultured bacteria and archaea using 16S rRNA gene sequences. Nat Rev Microbiol. 2014;12:635–45.
