# Supplementary material for: A ubiquitous subcuticular bacterial symbiont of a coral predator, the crown-of-thorns starfish, in the Indo-Pacific
Source: Microbiome. 2020 Aug 24;8:123. doi: 10.1186/s40168-020-00880-3 (PMC7444263; doi:10.1186/s40168-020-00880-3)
Supplement: Supplementary file 7 — Additional file 6: Supplementary materials and methods. [file 40168_2020_880_MOESM6_ESM.pdf]

SUPPLEMENTARY MATERIALS & METHODS

A universal subcuticular bacterial symbiont of a coral predator, the crown-of-thorns starfish

Naohisa WADA, Hideaki YUASA, Rei KAJITANI, Yasuhiro GOTOH, Yoshitoshi OGURA, Dai YOSHIMURA, Atsushi TOYODA, Sen-Lin TANG, Yukio HIGASHIMURA, Hugh SWEATMAN, Zac FORSMAN, Omri BRONSTEIN, Gal EYAL, Nalinee THONGTHAM, Takehiko ITOH, Tetsuya HAYASHI, Nina YASUDA

Sample collections and preparations

Six adult crown-of-thorns starfishes (COTSs) were used for 16S rRNA metabarcoding, three collected in Okinawa on Jul. 2017 and three in Miyazaki on Nov. 2017 (**Suppl. table S1**). The individuals were dissected into eight body parts (tips and bases of aboral spines from discs and arms, ambulacral spines, tube feet and pyloric stomachs, **Fig. 1**). Each body part was prepared primarily as triplicate subsample sets. One litter of sea water was also collected at each location at same time (three samples at each location), and filtered on a 0.2 μm filter (Millipore, USA). A total of 136 samples of body parts and filtered seawater were stored at -20˚C until DNA extraction. For the phylogenetic analysis based on the full-length 16S rRNA gene sequences, the tube feet of five individuals (n = 2 from Okinawa and n=3 from Miyazaki) were used.

The 195 COTS individuals used for PCR screening and sequencing of COTS27 included ethanol-preserved laboratory stocks, and those from our previous studies [1,2] collected between 2004 and 2017 (for more details see **Suppl. table S1**).

The COTS individual used for hologenome sequencing was collected in Miyazaki on Aug. 2014, and its tube feet samples were prepared and stored for DNA extraction in the modified CHAOS solution (4M guanidine thiocyanate, 0.1% N-lauroyl sarcosin sodium, 10 mM Tris pH8, 0.1 M 2-mercaptoethanol [3,4].

All DNA samples, except for the hologenome sequencing sample, were extracted using a protocol previously described [5] and dissolved in the TE (Tris-EDTA) solution for subsequent analyses. The genomic DNA for hologenome sequencing was extracted using the method described by Fukami et al. (2004).

Three adult individuals used for fluorescence *in situ* hybridization (FISH) analyses were collected in Miyazaki (Japan) on Apr. 2017 and dissected into six body parts: aboral spines from

both of disc and arms, tube feet, pyloric stomach, pyloric caeca and gonads (**Fig. 1b**). Each sample was fixed immediately in 4% paraformaldehyde phosphate buffer solution (Wako, Japan) for eight hours, and stored in 50% ethanol. The samples stored in 50% ethanol were rinsed with 100 mM phosphate-buffered saline (pH 7.4; NIPPON GENE, Japan) for three times, and then decalcified by the Morse's solution [6], dehydrated through ethanol gradient series (70% - Abs.), and embedded in paraffin according to the standard protocol.

Phylogenetic analysis of COTS27 using full-length 16S rRNA gene sequences

To determine the full-length 16S rRNA gene sequences of COTS27 we first designed two COTS27-specific primers, COTS_V4_R and COTS_V4R_F (**Suppl. fig. S1** and **Suppl. table S2**), based on the sequence of OTU1 using Primer-BLAST [7]. Analyses using the Silva SSU 132 database (https://www.arb-silva.de/) and TestPrime [8] confirmed that these primers can discriminate COTS27 from other bacterial sequences.

Next, the 16S rRNA gene was amplified by PCR using two primer sets (27F / COTS_V4_R and COTS_V4R_F / 1492R(c)), and two PCR products were directly sequenced from both directions. PCR amplifications were carried out in a 10 µl reaction mixture containing 1 µl of template DNA, 3.86 µl of dH_2O, 0.07 µl of each primer (50 µM), and 5 µl of KAPA Taq ReadyMix (Nippon Genetics Co. Ltd, Japan). The thermocycling program consisted of an initial denaturation at 95˚C for 2 min, 40 cycles of 94˚C for 30 sec, 50˚C for 30 sec and 72˚C for 90 sec, and a final extension at 72˚C for 5 min. The two PCR products were sequenced using the Big Dye Terminator Sequencing kit using the PCR primers on an ABI 3730 capillary sequencer (Applied Biosystems Inc., USA).

To close the sequencing gap between the two abovementioned amplicons, we designed another primer set, Microbiont F and R (**Suppl. fig. S1** and **Suppl. table S2**), and used another PCR

amplification for that gap region. PCR amplifications were performed in a 10 µl reaction mixture as described above. The thermocycling was performed with an initial denaturation at 94˚C for 1 min, 40 cycles of 94˚C for 20 sec, 55˚C for 45 sec and 72˚C for 3 min, and a final extension for 10 min at 72 ˚C. The PCR products were sequenced as described above.

COTS27 sequences were assembled using ATSQ software (GENETYX, Japan) to reconstruct full length 16S rRNA gene sequences. We retrieved all sequences of the phylum *Spirochaetes* available in the Living Tree Project (LTP) release 128 [9]. We also obtained additional reference sequences showing same cluster with COTS27 sequences in ARB software package [10] with the SSURef_NR99_128 database (https://www.arb-silva.de/). The determined sequences were aligned with all reference sequences using MUSCLE [11]. Phylogenetic trees were constructed using the Maximum likelihood (ML) method with the generalized time-reversible model with gamma distribution and proportion of invariable sites and the Neighbor-Joining (NJ) method, each applying 1000 bootstrap replications in MEGA7 [12].

PCR screening and sequencing of COTS27

Two PCR primers (COTSsymb F and R; **Suppl. fig. S1** and **Suppl. table S2**) were designed using the Primer-BLAST [7] to specifically amplify a 261-bp region of the COTS27 16S rRNA genes region. The specificity of these primers was confirmed as described above. An *Asteroidea*-universal primer set (hitode_16S f & r; **Suppl. fig. S1** and **Suppl. table S2**) that amplifies mitochondrial 16S rRNA gene sequence was also designed and used as a positive control for PCR reactions. PCR amplifications were performed in a 10 µl reaction mixture containing 1 µl of the genomic DNA, 3.86 µl of dH_2O, 0.07 µl of each primer (50 µM) and 5 µl of KAPA Taq ReadyMix (Nippon Genetics Co. Ltd). In several cases, KAPA2G Robust HotStart ReadyMix (Nippon Genetics Co. Ltd, Japan) or Go

Taq master mix (Takara, Japan) was used instead of KAPA Taq ReadyMix. PCR products were detected by electrophoresis on a 1% agarose gel. Selected PCR products (n=53) were sequenced using the COTSsymb F and R primers and used for constructing phylogenetic trees as described above.

Hologenome sequencing analysis

Reconstruction of the COTS27 chromosome sequence: Two paired-end (PE) libraries (insert sizes; 300 bp and 500 bp) were prepared and sequenced using the Illumina HiSeq 2500 sequencer. *De novo* assembly was performed using Platanus v. 1.2.3 [13]. To identify the COTS27-derived sequences, we extracted long scaffolds (≥5000 bp) that had higher depths of coverage (≥200×) compared to the average of all scaffolds (130×). Coverage depths were estimated using Platanus. In addition to the PE libraries, we prepared and sequenced six mate-pair (MP) libraries (insert sizes; 3, 5, 8, 10, 12, and 15 kb). Using all MP reads, additional scaffolding was performed for the potentially COTS27-derived scaffolds. As the longest scaffold was suspected as the COTS27 chromosome, several gaps in the scaffold were closed by PCR and Sanger sequencing as well as *in silico* based on the assembly results obtained using another assembler, Platanus-allee v. 2.0.0 [14]. The completeness of the obtained sequence was estimated by Check M [15].

Gene prediction and functional annotation: Gene prediction and functional annotation were performed using PROKKA v. 1.12 [16]. Predicted genes were manually confirmed utilizing In Silico Cloning Genomic Edition v. 5 (In Silico Biology Inc., Yokohama, Japan). Annotated gene names were curated using the following sources of information: (1) Blast hit table for the National Center for Biotechnology Information (NCBI) NR database and UniProt Knowledgebase (UniProtKB) Swiss-Prot, (2) protein information in UniProtKB, (3) protein signatures detected using InterProScan

v. 5.22-61.0 (Jones et al. 2014), (4) KEGG assignments of proteins by BlastKOALA v. 2.1 and KofamKOALA v. 2019-04-06, (5) operon information for *Escherichia coli* K-12 in the RegulonDB [17], and (6) membrane-related characteristics of proteins predicted using SOSUI version 1.10 [18].

COG assignments were obtained following the Joint Genome Institute (JGI) Microbial Genome Annotation Pipeline [19]. Using the COG, Position-Specific Scoring Matrices (PSSMs) obtained from Conserved Domains Database (CDD), genes were classified according to COG functional categories using RPS-BLAST (top hit, e-value cutoff of 1e-2, alignment length of at least 70% of the consensus sequence length). To obtain metabolic pathway information, K numbers were assigned using BlastKOALA and KofamKOALA. BlastKOALA was first used to search in the KEGG GENES database (selected taxonomic group, Bacteria; selected database, "species_prokaryotes"). COTS27 Genes that were not assigned by BlastKOALA were subjected to search in the KOfam database using KofamKOALA.

Comparison with other Spirochetes in the IMG database: A total of 834 Spirochetes genomes with COG annotation assigned were obtained from the IMG database. To select high-quality genome data, the 834 genomes were evaluated by CheckM using the *Spirochaetes* gene markers, and medium- or low-quality genomes (≤90% completeness and ≥5% contamination) were eliminated according to Bowers *et al.* 2017. Finally, 716 were retained as high-quality genome data (see **Suppl. Materials and Methods Fig. 1**) and used for the comparison with COTS27. PCA was performed using COG functional categories by the prcomp command and maptools pakage ver. 0.9-5 [20] in R [21].

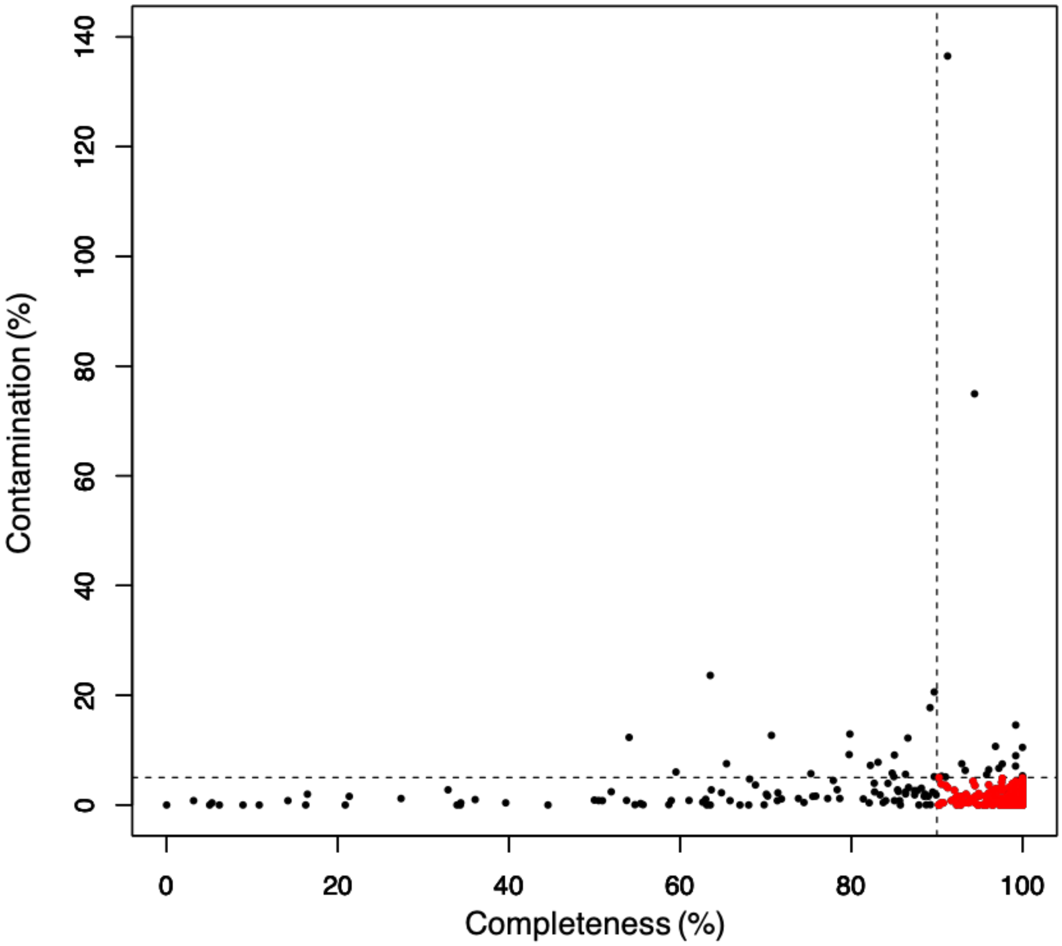

Suppl. Materials and Methods Fig. 1 Scatter plot of CheckM assignments of 834 Spirochaetes genomes obtained from the IMG database. Vertical and horizontal dashed lines correspond to 90% completeness and 5 % contamination, respectively. Red dots indicate the 716 high-quality genomes showing > 90% completeness and < 5% contamination.

References

1. Yasuda, N. *et al.* Gene flow of Acanthaster planci (L.) in relation to ocean currents revealed by microsatellite analysis. *Mol. Ecol.* **18**, 1574–1590 (2009).
2. Yasuda, N. *et al.* Latitudinal differentiation in the reproduction patterns of the crown-of-thorns starfish Acanthaster planci through the Ryukyu Island Archipelago. *Plankton Benthos Res.* **5**, 156–164 (2010).
3. Sargent, T. D., Jamrich, M. & Dawid, I. B. Cell interactions and the control of gene activity during early development of Xenopus laevis. *Dev. Biol.* **114**, 238–246 (1986).

4. Fukami, H. *et al.* Geographic Differences in Species Boundaries Among Members of the Montastraea Annularis Complex Based on Molecular and Morphological Markers. *Evolution* **58**, 324–337 (2004).
5. Yasuda, N., Taquet, C., Nagai, S., Yoshida, T. & Adjeroud, M. Genetic connectivity of the coral-eating sea star Acanthaster planci during the severe outbreak of 2006–2009 in the Society Islands, French Polynesia. *Mar. Ecol.* **36**, 668–678 (2015).
6. Morse, A. Formic Acid-Sodium Citrate Decalcification and Butyl Alcohol Dehydration of Teeth and Bones for Sectioning in Paraffin. *J. Dent. Res.* **24**, 143–153 (1945).
7. Ye, J. *et al.* Primer-BLAST: A tool to design target-specific primers for polymerase chain reaction. *BMC Bioinformatics* **13**, 134 (2012).
8. Klindworth, A. *et al.* Evaluation of general 16S ribosomal RNA gene PCR primers for classical and next-generation sequencing-based diversity studies. *Nucleic Acids Res.* **41**, e1 (2013).
9. Yarza, P. *et al.* The All-Species Living Tree project: A 16S rRNA-based phylogenetic tree of all sequenced type strains. *Syst. Appl. Microbiol.* **31**, 241–250 (2008).
10. Ludwig, W. *et al.* ARB: a software environment for sequence data. *Nucleic Acids Res.* **32**, 1363–1371 (2004).
11. Edgar, R. C. MUSCLE: multiple sequence alignment with high accuracy and high throughput. *Nucleic Acids Res.* **32**, 1792–1797 (2004).
12. Kumar, S., Stecher, G. & Tamura, K. MEGA7: Molecular Evolutionary Genetics Analysis Version 7.0 for Bigger Datasets. *Mol. Biol. Evol.* **33**, 1870–1874 (2016).
13. Kajitani, R. *et al.* Efficient de novo assembly of highly heterozygous genomes from whole-genome shotgun short reads. *Genome Res.* **24**, 1384–1395 (2014).
14. Kajitani, R. *et al.* Platanus-allee is a de novo haplotype assembler enabling a comprehensive access to divergent heterozygous regions. *Nat. Commun.* **10**, 1702 (2019).
15. Parks, D. H., Imelfort, M., Skennerton, C. T., Hugenholtz, P. & Tyson, G. W. CheckM: assessing the quality of microbial genomes recovered from isolates, single cells, and metagenomes. *Genome Res.* **25**, 1043–1055 (2015).
16. Seemann, T. Prokka: rapid prokaryotic genome annotation. *Bioinformatics* **30**, 2068–2069 (2014).
17. Gama-Castro, S. *et al.* RegulonDB version 9.0: high-level integration of gene regulation, coexpression, motif clustering and beyond. *Nucleic Acids Res.* **44**, D133–D143 (2016).
18. Hirokawa, T., Boon-Chieng, S. & Mitaku, S. SOSUI: classification and secondary structure prediction system for membrane proteins. *Bioinforma. Oxf. Engl.* **14**, 378–379 (1998).
19. Huntemann, M. *et al.* The standard operating procedure of the DOE-JGI Microbial Genome Annotation Pipeline (MGAP v.4). *Stand. Genomic Sci.* **10**, (2015).

20. Bivand, R. & Lewin-Koh, N. maptools: Tools for reading and handling spatial objects. *R Package Version 08* **23**, (2013).
21. R Development Core Team. *R: A Language and Environment for Statistical Computing*. (R Foundation for Statistical Computing, 2008).
